# Ir Single Atom Catalyst Loaded on Amorphous Carbon Materials with High HER Activity

**DOI:** 10.1002/advs.202105392

**Published:** 2022-03-09

**Authors:** Chunxiang Liu, Ganghuo Pan, Nianjie Liang, Song Hong, Jingyuan Ma, Yuzhou Liu

**Affiliations:** ^1^ School of Chemistry Beihang University Beijing 100191 China; ^2^ Center for Instrumental Analysis Beijing University of Chemical Technology Chaoyang Beijing 100029 China; ^3^ Shanghai Synchrotron Radiation Facility Shanghai Institute of Applied Physics Chinese Academy of Sciences Shanghai 201204 China; ^4^ Beijing Advanced Innovation Center for Biomedical Engineering Beihang University Beijing 100191 China

**Keywords:** 2D porous carbon material, hydrogen evolution reaction (HER), nanometer openings, single‐atom catalysis, water decomposition

## Abstract

The research of high efficiency water splitting catalyst is important for the development of renewable energy economy. Here, the progress in the preparation of high efficiency hydrogen evolution reaction (HER) catalyst is reported. The support material is based on a polyhexaphenylbenzene material with intrinsic holes, which heals into carbon materials upon heating. The healing process is found to be useful for anchoring various transition metal atoms, among which the supported Ir Single‐atom catalyst (SAC) catalyst shows much higher electrocatalytic activity and stability than the commercial Pt/C and Ir/C in HER. There is only 17 mV overpotential at 10 mA cm^–2^, which is significantly lower than that of commercial Pt/C and Ir/C catalysts respectively by 26 and 3 mV, and the catalyst has an ultra‐high mass activity (MA) of 51.6 ${\;A\;\text{mg}}_{\mathrm{Ir}}^{-1}$ at 70 mV potential and turn over frequencies (TOF) of 171.61 s^–1^ at the potential of 100 mV. The density functional theory (DFT) calculation reveals the significant role of carbon coordination around the Ir center. A series of monatomic PBN‐300‐M are synthesized by using of designed carbon materials. The findings provide an enabling and versatile platform for facile accessing SACs toward many industrial important reactions.

## Graphical abstract

A series of monatomic PBN‐300‐M are synthesized by use of designed carbon materials. By optimizing preparation conditions including the calcination temperature, PBN‐300‐Ir exhibits an overpotential as low as 17 mV (10 mA cm^−2^) in hydrogen evolution reaction with good stability. DFT calculation reveals the importance of carbon coordination for the high activity of PBN‐300‐Ir along with XANES analysis.

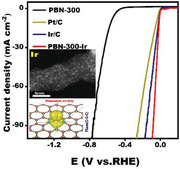

## 1 Introduction

Hydrogen (H_2_), as a significant part of regenerative clean energy sources, is expected to substitute the reduced fossil fuels in the future. High hydrogen production efficiency and low cost are the keys to realize the industrialization of hydrogen energy.^[^ ^1^ ^]^ HER, as one of many hydrogen production methods, has the advantages of cleanness and sustainability.^[^ ^2^, ^3^, ^4^ ^]^ Precious metals such as Pt and Ir can provide good HER activity, but the high cost and low content in the earth are the principal factors hindering their industrialization. SACs can maximize the utilization of metal atoms, and it is expected to be an effective way to industrialize precious metals economically and efficiently.^[^ ^5^, ^6^, ^7^, ^8^, ^9^, ^10^ ^]^

Many ways have been developed for preparation of SACs, but the substrates usually lack the good conductivity for facilitating electron transportation. Carbon materials, with its peculiar two‐dimensional conjugated structure,^[^ ^11^ ^]^ can be ideal candidates for such purpose^.[^ ^12^ ^]^ However, due to the absence of lone‐pair electrons in pristine carbon materials, the supported metal atoms exhibited relatively weak interaction with the carbon materials surface, and always aggregated into large particles with reduced mass activities. How to effectively anchor catalytic centers on an all‐carbon conjugated framework, preferably at the single‐atom level, is one key problem, and the solving of which could lead to electrocatalysts with very high MA and TOFs.

Inspired by previous report that the nanometer defects can be repaired by C—C bond reconstruction and has the ability to capture fixed single atoms,^[^ ^7^, ^13^, ^14^ ^]^ we sought to prepare such materials in bulk. Herein, we would like to report our work in bulk synthesis of a 2D‐carbon materials derivative with nanometer holes by bottom‐up approach. This 2D conjugated carbon framework could not only self‐heal, but also formed C—M bonds and C‐coordinated well‐distributed monatoms when various volatile metal precursors (M═Fe, Co, Ni, Mn, Pd, Mo, W, Re, Ir) were introduced. Among them, the single‐atom Ir catalyst exhibited HER efficiency one order higher than previously the best ones, while also possessed very high stability. Our work represents a new way of fabricating single‐atom metal catalysts in 2D Carbon materials with high catalytic efficiencies.

## 2 Results and Discussion

### 2.1 Synthesis and Structural Characterization

**Figure 1:**
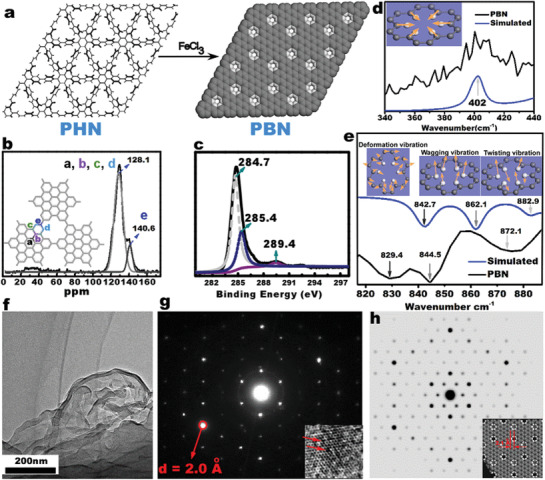
The characterizations of the nanometer hole defects on PBN. a) The reaction scheme to prepare PBN, b) Solid state ^13^C NMR spectrum, c) C 1s XPS spectrum, d) Raman spectrum, e) IR spectrum, f) TEM image, g) HRTEM image with SAED spectrum (inset), h) Layered offset stacking structure of PBN with holes on top of hexabenzocoronene cores (inset: the electron diffraction pattern of the simulated structure).

Our bottom‐up bulk synthesis was shown in **Figure** **1**a, in which the substitution of six adjacent carbon atoms in carbon skeleton by hydrogen atoms would generate a carbon material derivative with nanometer holes and hexabenzocoronene subunits. As shown in Figure 1a, the designed holey carbon skeleton structure can be easily prepared in bulk through simple oxidation of a topologically equivalent polyhexaphenylbenzene network (PHN). Upon being soaked in the CH_2_Cl_2_ solution of FeCl_3_ at room temperature, white PHN powder turned into dark brown immediately, which indicated the occurrence of the cyclodehydrogenation reaction on the hexaphenylbenzene units.

The formation of crystalline FeCl_2_, confirmed by X‐ray diffraction (Figure S1, Supporting Information) also indicated the occurrence of the cyclodehydrogenation reaction. The full conjugation after cyclodehydrogenation then topologically led to the formation of the holely polyhexabenzocoronene network (PBN), which was supported by multiple analyses.

Solid‐state ^13^C‐NMR spectrum (Figure 1b) showed expected high‐field movements of the C atoms in PBN compared to PHN (Figure S2, Supporting Information) due to the formation of conjugated hexabenzocoronene pieces, and the integral ratio between the low‐field (d) and high‐field signals (a, b, c, e) in Figure 1b matched the expected value (1:6.3 vs 1:6 respectively), consistent with the full conversion of the hexaphenylbenzene units into hexabenzocoronene cores. The XPS shake‐up satellite peak for C (1s) (Figure 1c) at 289.4 eV (due to carbon *π* to π* transition) has been reported as a sign of long‐range conjugation,^[^ ^15^ ^]^ and the appearance of such peak in PBN, not in PHN (Figure S3, Supporting Information) or any reported isolated hexabenzocoronene molecules, also clearly supported the 2D conjugated nature in PBN. The Raman spectrum of PBN showed the expected features of similar graphitic molecules, namely the D and G bands, which were completely absent in PHN (Figure S4, Supporting Information), and this supported the successful formation of conjugated hexabenzocoronene cores.

The formation of PBN was also supported by appearance of Raman peak at 402 cm^–1^ and infrared spectroscopy (IR) peak at 844.5 cm^–1^_,_ both of which have been known as markers for such holes.^[^ ^16^, ^17^ ^]^ The 402 cm^–1^ Raman marker (Figure 1d, Figure S5, Supporting Information) has been reported to originate from a collective contraction along the C—H bonds surrounding the hole, which was actually confirmed through density functional theory (DFT) calculation of the PBN model shown in Figure 1a (see S3‐2 for details, Figure S6, Supporting Information). DFT calculation also corresponded the experimental IR bands at 872.1, 844.5, and 829.4 cm^−1^ (Figure 1e) to the twisting, wagging and deforming vibrations.^[^ ^18^, ^19^ ^]^ Overall, these spectroscopic characterizations provided strong supporting evidences for the existence of such nanometer holes.

TEM imaging (Figure 1f) revealed the sheet‐like morphology, and aberration‐corrected high‐angle annular dark‐field scanning TEM (ACHAADF‐STEM) and HRTEM of a crystalline region (inset of Figure 1g) led to direct viewing of the openings at the single‐atom level. The structure of PBN was also supported by electron diffraction under HRTEM imaging, in which the selected area electron diffraction pattern (SAED) (Figure 1g) matched perfectly with the predicated one (Figure 1h) based on the offset stacking model (atomic coordinates for the model in S3, Supporting Information) in terms of positions of major peaks and relative intensity.

The [−3, −3, 0] set Miller planes were clearly revealed in SAED (red circle in Figure 1g). The relative plane distance, representing roughly half width of the hole, was around 2.0 Å calculated from SAED pattern and 2.1 Å based on the theoretical model. The good matching strongly supported the formation of similar graphene structure with hole defects and also the phase purity of PBN. Our structure was similar to a polyphenyl network with similar holes.^[^ ^15^ ^]^

Interestingly, heating PBN at 300 °C for 7 h in argon led to the complete disappearance of the band gap (Figure S8, Supporting Information) with the formation of a black and conducting carbon material at room temperature (named as PBN‐300, for conductance measurement). This indicated the loss of these holes during the 300 °C heating (Figures S9 and S10, Supporting Information). We speculated that the loss of CH groups would lead to momentary formation of carbon defects, which then self‐healed to form a conjugated carbon network (Figure S11, Supporting Information). We expected that the conductive substrate can reduce the barrier of electron transfer and improve the kinetic performance of the catalyst.

### 2.2 Analysis of Composition and Atomic Structure

**Figure 2:**
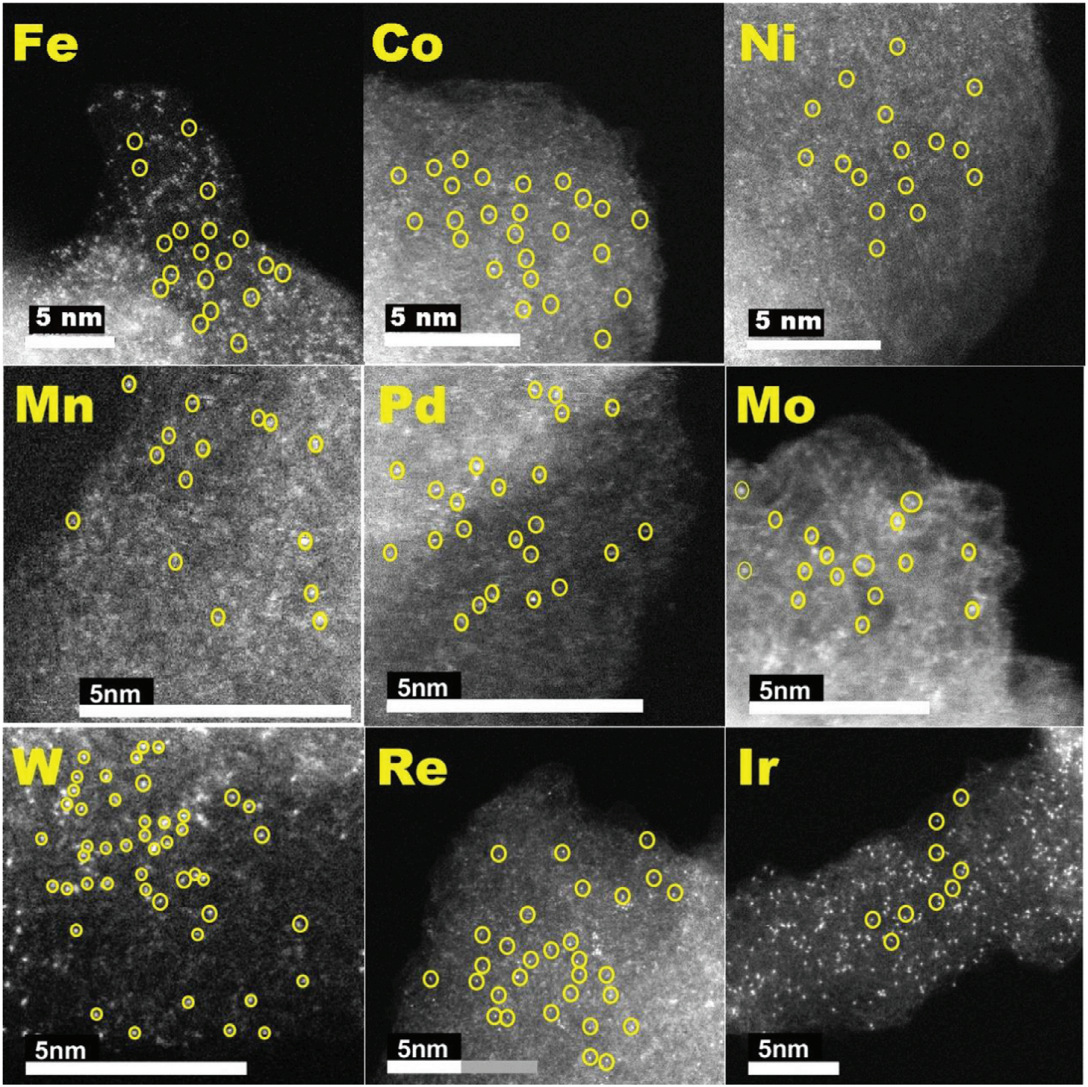
ACHAADF‐STEM imaging of PBN‐300‐M (M═Fe, Co, Ni, Mn, Pd, Mo, W, Re, Ir) samples. The atomic distribution of various metal elements was evident.

**Figure 3:**
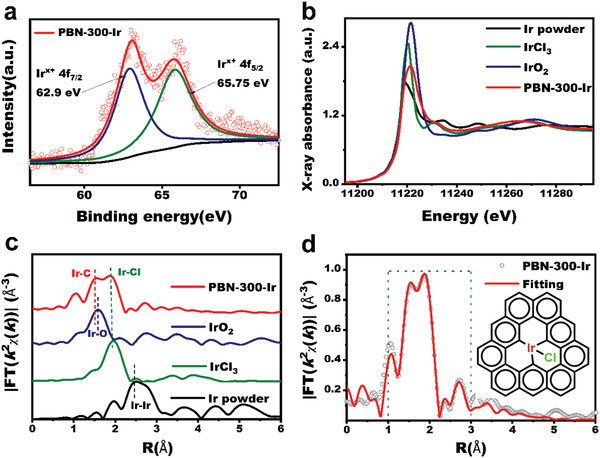
Morphology and structure characterization of PBN‐300‐Ir. a) XPS imaging of PBN‐300‐Ir samples. b) XANES of Ir L3‐edge, where the white line intensity shows the valance state of Ir in PBN‐300‐Ir is between +1 and +3. c) Fourier transforms of k2‐weighted Ir L3‐edge EXAFS data; d) the total fit signal (red line) is superimposed on the experimental signal of Ir L3‐edge EXAFS of PBN‐300‐Ir (gray‐cycle). The measured and calculated spectra show excellent agreement.

Volatile metal precursors (M^x+^, M = Ir/Fe/Co/Ni/Pd/Mn/Mo/W/Re) were added prior to heating with the aim of incorporating metal atoms in the carbon matrix respectively. We expected that momentary carbon defects could capture these metal atoms to form C—M bonds.^[^ ^20^, ^21^ ^]^ In the pyrolysis process under 300 °C and argon atmosphere, PBN precursor was transformed into carrier with defects. At the same time, the coordinated M^x+^ ion was reduced to a metal single atom (M_1_) through the carbonization of the organic linker, thereby forming an isolated M embedded in the carrier PBN (PBN‐300‐M). The uniform distribution of Ir atoms in PBN‐300‐Ir was clearly evidenced by EDX analysis during TEM imaging, and no Ir atom aggregations were detected even at low resolution. Aberration‐corrected high‐angle annular dark‐field scanning TEM (ACHAADF‐STEM) revealed the Ir element was present at the single‐atom level as shown in **Figure** **2** (The content of Ir element in PBN‐300‐Ir was around 0.74%, estimated by ICP‐OES (Figure S12, Supporting Information)). The mapping of the HAADF image in Figure S12 (Supporting Information) demonstrated that the Ir atoms were evenly distributed on the PBN substrate. The single‐atom distribution of Ir atom in the sample was further confirmed by X‐ray adsorption spectroscopy (XAS) (**Figure** **3**b,c), which showed the absence of M—M bonds in all these samples.^[^ ^22^ ^]^ It is considered that the PBN‐300‐Ir can display geometric and electronic structures differ in those of the single atoms that are produced by fixing on the carrier.^[^ ^23^, ^24^ ^]^

More remarkably, based on the above C–M coordination interaction, this preparation route can be extended to a variety of transition metals, including Fe， Co， Ni， Pd， Mn， Mo， W， Re. The absence of metal particles and aggregates in the resulting material was confirmed by HRTEM with EDS mapping, as shown in Figure S13 (Supporting Information), these single atoms of Fe, Co, Ni, Mn, Mo, Pd, Re, and W were successfully mixed on PBN in a manner analogue to Ir without birdnesting. We also measure the Pd/W/Re/Mo/Fe/Mn content by EDS, which was around 3.4%, 0.7%, 6.9%, 0.3%, 2%, 0.24% by mass (Figures S14 and S15, Supporting Information). The results of EDS show that the loading contents of Pd， W， Re， Mo， Fe， Mn on PBN are significantly different, compared with 0.74% of PBN‐300‐Ir. According to the above results, the process of PBN stabilizing atoms may be similar to the process of graphene defect capturing atoms. When the atoms around the hole were activated, the metal atoms in the environment were captured to form an all carbon coordinated monatomic catalyst.^[^ ^21^, ^25^ ^]^ In addition, as shown in (Figure 2), HAADF‐STEM images exhibit that the single atom of platinum Fe, Co, Ni, Mn, Pd, Mo, W, Re, and Ir is separated and fixed on the C substrate, indicating a very strong coordination between C and M. XAS analysis of these samples confirmed the absence of M—M bonds (Figure S16, Supporting Information). These clearly indicated that PBN was able to trap transition metal atoms with quite a good generality when volatile metal complex were present during heating.

The electronic state of PBN‐300‐Ir with unique structure is obviously different to that of IrO_2_ and Ir powder. We studied the electronic valence state of Ir in PBN‐300‐Ir by EXAFS and X‐ray photoelectron spectroscopy (XPS). As can be seen from Figure 3a, in PBN‐300‐Ir, the 65.75 eV refers to the Ir4f_5/2_ peak and 62.9 eV is related to the Ir 4f_7/2_ peak. The Ir 4f_5/2_ (65.75 eV) of PBN‐300‐Ir is 0.2 eV lower than that of IrO_2_ (65.90 eV).^[^ ^26^ ^]^ It indicates that the Ir valence state of PBN‐300‐Ir is lower than Ir^4+^. Ir 4f_5/2_ of PBN‐300‐Ir is higher than 4f_5/2_ (63.8 eV) of metallic Ir.^[^ ^27^ ^]^ It is further proved that the valence state of Ir in PBN‐300‐Ir is higher than Ir^0^. It matches with XANES spectra of Ir L3‐edge. As show in Figure 3b, in XANES spectra of Ir L3‐edge, Ir powder has the lowest white line intensity and the peak intensity of PBN‐300‐Ir is between Ir powder and IrCl_3_, and this indicates that its Ir is partially oxidized in the state of Ir ^x+^. Further attention was paid to the immediate environment around the Ir centers in PBN‐300‐Ir through X‐ray Absorption Energy Near edge Structure (XANES) spectroscopy and extended X‐ray absorption fine structure (EXAFS) (Figure 3b,c). Commercial Ir powder, IrCl_3_, IrO_2_ were employed as benchmarks for identification of the Ir—Ir, Ir—O, and Ir—Cl bonds (Figures S17 and S18, Supporting Information).

In the Fourier transformation (FTs, R‐space, Figure 3c) for EXAFS, the spectrum of PBN‐300‐Ir has two main peaks of ≈1.56 and ≈1.97 Å. No Ir−Ir coordination was found, and the main peak of Ir powder was ≈2.7 Å (Table S1, Supporting Information). There is no Ir–Ir scattering in PBN‐300‐Ir spectrum, which strongly confirms its atomic isolation. The peak of 1.97 Å is clearly visible in PBN‐300‐Ir and IrCl_3_, indicating the presence of Ir—Cl bond in PBN‐300‐Ir. This result matches the XPS result, which showed the presence of Ir—Cl bonds. In addition, The radial distance resolution and k‐space resolution of EXAFS wavelet transform (WT) were used to further identify the monatomic Ir.^[^ ^28^ ^]^ According to FT analysis, there are no metal particles in PBN‐300‐Ir. PBN‐300‐Ir has the maximum value of 6.2 Å^–1^. By contrast, in IrO_2_ and Ir powders, strength maxima appear at higher k‐spaces 8.7 (Ir–O) and 10.5 (Ir–Ir) (Figure S19, Supporting Information), respectively. Quantitative least squares EXAFS curve‐fitting analysis (Figures S20, S21 and Table S2, Supporting Information) suggested the square‐pyramidal configuration (Figure 3d) with the coordination numbers of 3.5 and 1.0 for C and Cl atoms respectively. The embedding of the Ir atoms inside the carbon layer prevented the formation of Ir particles, which usually happens for Ir atoms supported by defect‐free carbon material.^[^ ^29^ ^]^ The above analysis results support that the coordination structure formed in PBN‐300‐Ir.

### 2.3 Electrochemical Analysis

**Figure 4:**
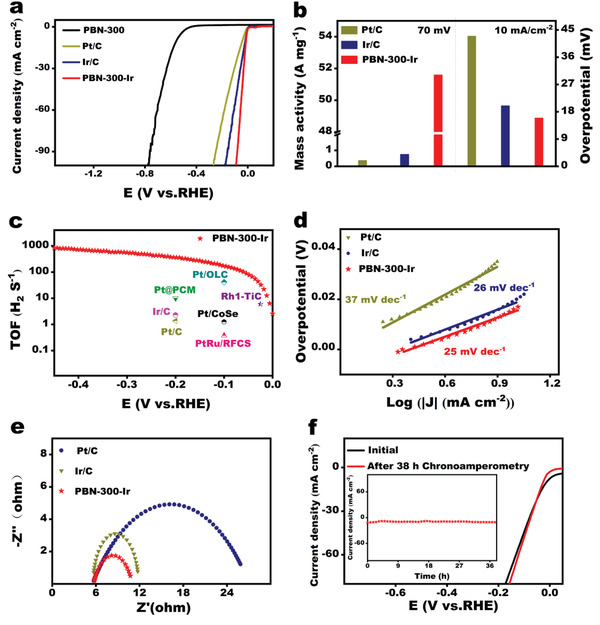
Electrocatalytic properties. a) The HER polarization curves for PBN‐300‐Ir, PBN‐300, Pt/C, and Ir/C were acquired by linear sweep voltammetry with a scan rate of 5 mV s^–1^ in 0.5 m H_2_SO_4_ at room temperature. N_2_ was purged before the experiments. b) Comparison between the mass activity of PBN‐300‐Ir, Pt/C, and Ir/C at 70 mV and the overpotentials required to achieve 10 mA cm^–2^ for PBN‐300‐Ir, Pt/C and Ir/C. c) TOF values of PBN‐300‐Ir, Pt/C, and Ir/C, together with previous reported HER electrocatalysts (Table S6, Supporting Information). d) Tafel plots for PBN‐300‐Ir, Pt/C and Ir/C. e) Electrochemical impedance spectroscopy data for PBN‐300‐Ir, Pt/C, and Ir/C using insert illustration. Data were collected for the electrodes under HER overpotential of 10 mV. f) Comparison of LSV curves before (black) and after (red) chronoamperometry test. The inset is current density versus time (*I*–*T*) curves of PBN‐300‐Ir recorded for 38 h at −0.019 V versus RHE.

The HER performance of PBN‐300‐Ir was then measured and compared to those of commercially available Ir/C and Pt/C materials. As shown in **Figure** **4**a, at the loading of 200 µg cm^–2^ (relatively 1.4 μg_Ir_ cm^–2^), PBN‐300‐Ir exhibited an overpotential of only 17 mV versus reversible hydrogen electrode (RHE) to achieve a 10 mA cm^–2^ HER current density in 0.5 m aqueous H_2_SO_4_ at room temperature. In contrast, commercial 5 wt% Pt/C (1000 µg cm^–2^, 50 μg_Pt_ cm^–2^) exhibited an overpotential of 43 mV at 10 mA cm^–2^ even with a much higher loading of Pt. Similarly, Ir/C (1000 µg cm^–2^, 50 μg_Ir_ cm^–2^) showed an overpotential of 20 mV at 10 mA cm^–2^. Figure S22 (Supporting Information) shows the representative polarization curves for HER under all pH values. PBN‐300‐Ir displayed a considerable activity, requiring an overpotential of 17, 33, and 83 mV to achieve the current density of −10 mA cm^–2^ for pH 0.35, 7, and 13.71, respectively. According to the influence of temperature and precursor Ir loading content on HER activity of PBN‐T‐Ir (Figures S23 and S24, Supporting Information), PBN‐300‐Ir has the best HER activity in 0.5 m H_2_SO_4_. Although the Ir content is low, PBN‐300‐Ir has higher Ir atom utilization productivity compared with 5 wt% Pt/C and Ir/C, which is certificated by MA calculation. The MA of the HER for PBN‐300‐Ir at the overpotential of 70 mV was remarkably as high as 51.6 ${\;A\;\text{mg}}_{\mathrm{Ir}}^{-1}$ (Figure 4b), which was around 139 times that of commercial Pt/C (0.37 ${\;A\;\text{mg}}_{\mathrm{Pt}}^{-1}$) and 68 times that of commercial Ir/C (0.75 ${\;A\;\text{mg}}_{\mathrm{Ir}}^{-1}$). It is clearly proved that the utilization productivity of Ir atoms in PBN‐300‐Ir is remarkably improved. As shown in Figure 4c, the TOF of HER for PBN‐300‐Ir at 100 mV was estimated to be around 170.61 s^–1^, ranking among the highest ones up to date (Table S3, Supporting Information).^[14,30]^ The extraordinary improvement on the MA and TOF is reflective of the single‐atom distribution of Ir in our case, maximizing the efficiency of each atom. The aforementioned results show that although the content of Ir in PBN‐300‐Ir is low, the activity is much better than that of commercial Pt/C and Ir/C. This significantly proves the important role of the electronic structure and coordination environment of Ir atom during the HER reaction.

The fast kinetics for PBN‐300‐Ir was also confirmed by the Tafel slope in Figure 4d, which was around 25 mV dec^–1^. The Tafel slope is a unique parameter of an electrocatalyst determined from its mechanism. The Tafel slope can be obtained from linear fitting between the linear portion of the polarization curve to the semilogarithmic equation *η* = *a* + *b*log *j*. The electrocatalytic activity can be determined from the slope and intercept of the Tafel plot, which is shown in Figure 4d. A small Tafel slope leads to good reaction kinetics for the HER and indicates a slower increase in overpotential with increasing current density.^[^ ^31^ ^]^ The fast kinetics for PBN‐300‐Ir was also confirmed by the Tafel slope in Figure 4d, which was around 25 mV dec^–1^. The dynamics nature of HER reaction for these cathodes was analyzed in Tafel slope diagram (Figure 4d). PBN‐300‐Ir has a smaller Tafel slope of 25 mV dec^–1^ compared with Pt/C (37 mV dec^–1^) and Ir/C (26 mV dec^–1^). The small Tafel slope indicated very low energy barrier, justifying the overall electrocatalytic activity. Since the Tafel slope value is about 30 mV dec^–1^, the HER process adopts the Volmer‐Tafel mechanisms, and the rate‐determining step is the Tafel reaction,^[^ ^32^ ^]^ in which adsorbed hydrogen atoms recombine to form hydrogen molecules. HER dynamics was expounded again by electrochemical impedance spectroscopy (EIS) (Figure 4e). The resistance of PBN‐300‐Ir is significantly smaller than that of Pt/C and Ir/C .The high activity was also consistent with the high conductance as measured by the electrochemical impedance spectroscopy. PBN‐300‐Ir acted out extraordinary stability in HER measurement.

The stability of PBN‐300‐Ir is evaluated by chronoamperometry (CA). The constant potential is −19 mV (vs RHE). It can be seen that when the working currnent density is 10 mA cm^−2^, the HER reaction is carried out continuously for more than 42 h, and the potential is basically unchanged (Figure S25, Supporting Information). Therefore, it can be seen that PBN‐300‐Ir has good stability. In Figure 4f, the current density of PBN‐300‐Ir after HER reaction in strong acid electrolyte for 38 h is basically unchanged. According to the LSV comparison before and after stability, the corresponding overpotential is basically the same under the working current density of 10 mA cm^–2,^ indicating that PBN‐300‐Ir has good stability. The HRTEM analysis of PBN‐300‐Ir after CA (Figure S26, Supporting Information) shows that there is no significant change on the surface of PBN. Ir was evenly dispersed on PBN without obvious agglomeration. It shows that the strong interaction of PBN with Ir enhances its stability.

**Figure 5:**
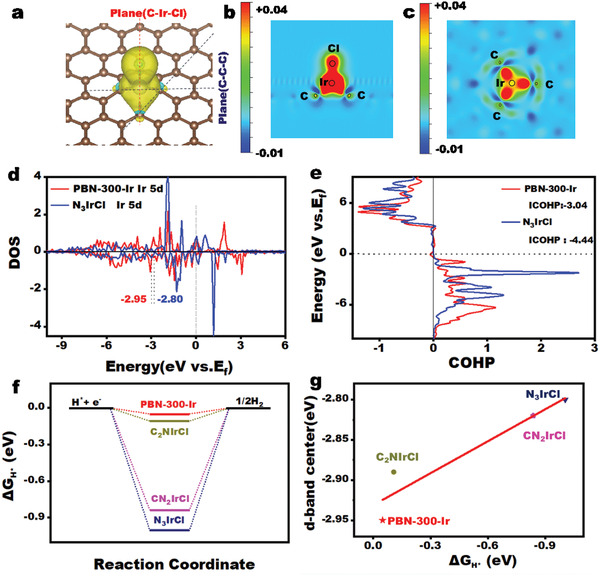
DFT calculations of PBN‐300‐Ir. a) The charge density difference diagram of PBN‐300‐Ir. b,c) The charge density difference diagrams along the (C–Ir–Cl) and (C–C–C) planes. d) DOS of PBN‐300‐Ir, N_3_IrCl. e) COHP of Ir for PBN‐300‐Ir and N_3_IrCl. f) Gibbs free energy of hydrogen adsorption on PBN‐300‐Ir, C_2_NIrCl, CN_2_IrCl, N_3_IrCl. g) Relationship between adsorptions energy of H and d‐band center of Ir of PBN‐300‐Ir, C_2_NIrCl, CN_2_IrCl, N_3_IrCl.

### 2.4 First‐Principles Calculations

We carried out the DFT calculations to further study the origin of the high HER activity of PBN‐300‐Ir by investigating the local environment of the Ir center. The C_3_IrCl model is constructed based on previous analysis (**Figure** **5a**). Figure 5b,c represented the charge density maps along the planes of (C–Ir–Cl) and (C–C–C) respectively. The red area indicates charge gained, and the blue area showed where the electrons were transferred to other atoms. According to the color diagram, here, we concluded that C obtained extra electrons from Ir. The electron transfer is also supported by Bader charge result analysis, in which the charge numbers of three C atoms around the Ir center are −0.077, −0.130, and −0.130, respectively. Therefore, the electron transfer from the Ir center to C atoms is confirmed, and the three adjacent carbon atoms are expected to participate significantly in the HER process, and be responsible for the high activity. Given the higher mass activity than most known N‐coordinated Ir catalysts,^[^ ^14^ ^]^ we further examined the importance of the carbon coordination in PBN‐300‐Ir, by comparing its electronic properties with N analogies. A series of C_x_N_y_IrCl structures are constructed and they represent those in which part of carbon atoms in PBN‐300‐Ir are replaced by nitrogen atoms (Figure S27, Supporting Information).

The partial density of states (PDOS) of the Ir 5d orbit (Figure 5d) in PBN‐300‐Ir and N_3_IrCl models were then calculated. Crystal orbital Hamilton population (COHP) (Figure 5e) of PBN‐300‐Ir was carried out. Figure 5d exhibits the PDOS values of the active centers Ir of PBN‐300‐Ir and N_3_IrCl along with the relevant d‐band center. The d‐band center of PBN‐300‐Ir is −2.93 eV far away the Fermi level, which is more negative than that of N_3_IrCl by −2.80 eV.^[^ ^33^ ^]^ The integral COHP value of Ir–H in PBN‐300‐Ir is −3.04 eV (Figure 5e), which is less than the relative values of Ir—H bond in N_3_IrCl (−4.44 eV), which further confirms the weak binding between Ir and H atoms on the active surface in PBN‐300‐Ir.^[^ ^34^ ^]^ These analysis shows the unique roles of coordinated C atoms around the Ir atoms, and they can facilitate the releasing of H_2_ from the absorption center, and therefore improving the efficiency of HER.^[^ ^35^ ^]^

As shown in Figure 5f, the minimum Gibbs free energy of hydrogen adsorption of PBN‐300‐Ir is –49 mV, showing a minimum energy well for the formation of hydrogen gas. If one or more of the carbon atoms around Ir center was substituted by nitrogen atoms, the feasibility to form H_2_ was significantly reduced (Table S3, Supporting Information). As shown in Figure 5g, the more negative the d‐band center is, the lower its adsorption energy for H, indicating that the d‐band center decreases, weakening the interaction between the catalytic center and the adsorbed hydrogen, thus weakening the reaction energy barrier of HER. The d‐low band center of Ir is conducive to the desorption of hydrogen,^[^ ^36^, ^37^, ^38^, ^39^ ^]^ and this explained the origin of the high reactivity of PBN‐300‐Ir.

## 3 Conclusion

In conclusion, PBN‐300‐Ir, a carbon coordination monatomic Ir catalyst supported by PBN, has high activity and long‐term stability in strongly acidic electrolyte. In 0.5 m H_2_SO_4_, the mass activity of PBN‐300‐Ir was 51.6 ${\;A\;\text{mg}}_{\mathrm{Ir}}^{-1}$ (70 mV), while the overpotential was only 17 mV (10 mA cm^–2^). The results from EIS further prove that PBN‐300‐Ir has low conductive barrier. The above results show that PBN‐300‐Ir has multiple active sites and low energy consumption. The Tafel slope of PBN‐300‐Ir is only 25 mV dec^–1^, which proves that it has good hydrogen evolution kinetic performance. DFT calculation shows that the d‐band center of PBN‐300‐Ir is lower than that of CNIrCl and the C–M structure of PBN‐300‐Ir reduces the adsorption energy of HER. Our new 2D carbon material showed unexpected and excitingly new properties, and it is expected that it will accommodate extended range of metal atoms and new applications are going to emerge for this interesting new material.

## Conflict of Interest

The authors declare no conflict of interest.

## Supporting information

Supporting information

## Data Availability

Research data are not shared.
