# Supplementary material for: Ir Single Atom Catalyst Loaded on Amorphous Carbon Materials with High HER Activity
Source: Adv Sci (Weinh). 2022 Mar 9;9(13):2105392. doi: 10.1002/advs.202105392 (PMC9069379; doi:10.1002/advs.202105392)
Supplement: Supplementary file 1 — Supporting information [file ADVS-9-2105392-s001.pdf]

ADVANCED SCIENCE
Open Access

Supporting Information

for *Adv. Sci.*, DOI 10.1002/advs.202105392

Ir Single Atom Catalyst Loaded on Amorphous Carbon Materials with High HER Activity

*Chunxiang Liu, Ganghuo Pan, Nianjie Liang, Song Hong, Jingyuan Ma and Yuzhou Liu**

Supporting Information

Ir single atom catalyst loaded on amorphous carbon materials with high HER activity

Chunxiang Liu , Ganghuo Pan , Nianjie Liang, Song Hong , Jingyuan Ma , Yuzhou Liu[*]

1. School of Chemistry, Beihang University, Beijing, 100191, China
2. Center for Instrumental Analysis, Beijing University of Chemical Technology, Chaoyang, Beijing 100029, China
3. Shanghai Synchrotron Radiation Facility, Shanghai Institute of Applied Physics Chinese Academy of Sciences, Shanghai, 201204, China
4. Beijing Advanced Innovation Center for Biomedical Engineering, Beihang University, Beijing, 100191, China

E-mail: liuyuzhou@buaa.edu.cn

S1 Materials and Methods

S1-1: Materials and Equipment

All materials were purchased from Innochem and used as received without further purification, unless otherwise noted. Ethanol was dried with Mg turnings.

All manipulations were carried out in air atmosphere, unless otherwise noted. The transmission electron microscopy (TEM and HRTEM) experiments were recorded on a Hitachi 7650 electron microscope. The IR spectra were recorded with KBr pellets on a Bruker EQUINOX 55 FT-spectrometer in the range of 4000−500 cm^{-1}. Powder X-ray diffraction (PXRD) patterns were recorded on a Bruker (D8 focus) analytical diffractometer for Cu Ka radiation (a = 1.5406 Å), with a scan speed of 5° min^{-1}. Raman spectra were recorded on a Lab RAM HR Evolution at 532 nm. The solid-state ^{13}C NMR spectra were done on a Varian VNMRS 600MHz. Atomic force microscope (AFM) images were captured on a Bruker Dimension Icon with Scan Asyst. Aberration-corrected high-angle annular dark-field scanning TEM (AC-HAADF-STEM) were performed by a JEOL JEM-ARM200F operating at 200 kV. X-ray photoelectron spectroscopy (XPS) was characterized using a Thermo Fisher ESCALAB 250XiPHI Quantera instrument equipped with an Al X-ray excitation source (1486.6 eV). Binding energies were corrected by reference to the C 1s peak at 284.8 eV. Elemental analysis (EA) were measured using FlashEA 1112. Cyclic voltammetry measurements were carried out in a standard one-compartment cell under atomsphere at 25 °C equipped with a carbon rob counter electrode, an SCE reference electrode, and a glassy carbon (GC) working electrode using a CHI760e Shanghai electrochemical analyzer. The measurements were performed in deionized water with 0.5 M H_2SO_4 as the supporting electrolyte. UV-vis-NIR absorption spectra were recorded on UV-3600 spectrometer. ICP-OES measurement was performed using Optima-7000 DV spectrometer. Hyphenation of TG-FTIR-GCMS was recorded at analytical Instrumentation Central of Peking University. XAFS measurement and data analysis: XAFS spectra at the Ir K-edge were collected at the beamline BL14W1 station of the Shanghai Synchrotron Radiation Facility (SSRF), China. Ir powder, $IrCl_3$, IrO_2 were used as references. The Ir K-edge XANES data were recorded in a fluorescence mode.

X-ray absorption fine structure spectroscopy

The acquired EXAFS data were processed according to the standard procedures using the ATHEN Amodule implemented in the IFEFFIT software packages[S1]. The k2-weighted EXAFS spectra were obtained by subtracting the post-edge background from the overall absorption and then normalizing with respect to the edge-jump step. Subsequently, k2-weighted x(k) data of Ir K-edge were Fourier

transformed to real (R) space using a hanning windows (dk=1.0 Å^{-1}) to separate the EXAFS contributions from different coordination shells. To obtain the quantitative structural parameters around central atoms, least-squares curve parameter fitting was performed using the ARTEMIS module of IFEFFIT software packages. The following EXAFS equation was used:

$$\chi(k) = \sum_j \frac{N_j S_o^2 F_j(k)}{k R_j^2} \exp[-2k^2 \sigma_j^2] \exp[\frac{-2R_j}{\lambda(k)}] \sin[2k R_j + \phi_j(k)]$$

S_0^2 is the amplitude reduction factor, $F_j(k)$ is the effective curved-wave back scattering amplitude, N_j is the number of neighbors in the jth atomic shell, R_j is the distance between the X-ray absorbing central atom and the atoms in the jth atomic shell (back scatterer), is the mean free path in Å, $\phi_j(k)$is the phase shift (including the phase shift for each shell and the total central atom phase shift), σ_j is the Debye-Waller parameter of the jth atomic shell (variation of distances around the average R_j). The functions $F_j(k)$and $\phi_j(k)$ were calculated with the ab initio code FEFF8.2.

Electrochemical measurements

All electrochemical measurements were performed on a CHI 760E workstation using a three-electrode cell equipped with a graphite rob as the counter electrode and an Ag/AgCl electrode (calibrated) and Hg/HgO as the reference electrode. HER measurements were respectively conducted in 0.5 M H_2SO_4， 0.1 M PBS and 1M KOH at room temperature. The reference electrode was converted to RHE according to the Nernst equation: (E_{RHE}= $E_{Ag/AgCl}$+ 0.0591 *pH+ 0.197) and (E_{RHE}= $E_{Hg/HgO}$+ 0.0591 *pH+ 0.098) . EIS spectra were performed with a frequency ranging from 10^6 Hz to 0.01 Hz and an amplitude of the sinusoidal voltage of 10 mV in the 0.5 M H_2SO_4 . The Chronoamperometry (CA) curves were obtained at a static overpotential. The current density–time curves was collected by loading catalyst ink onto carbon paper (1 cm × 1 cm) and the overpotential maintained at an η of 10 mV for 4 h. All LSV curves were not corrected. Chronopotentiometry(CP) test is based on the potential time curve when the constant current density is 10 mA cm^{-2}.

To fabricate the working electrode, 5 mg of each catalyst and electric conduction with 100 μl of 5 wt% Nafion solution (Sigma-Aldrich) was dispersed in 1 ml of a water/isopropanol mixed solvent (3:1 volume ratio) by 30 min of sonication to form a homogeneous ink. A glassy carbon disk served as the working electrode to drop the catalyst ink on the surface, which resulted in a catalyst loading of 200 μg cm^{-2} (calculated by the total weight) for all electrochemical tests. The glassy carbon electrode is 4 mm in diameter.

To prepare the Pt/C electrodes, 5.0 mg Pt/C powder (10 wt%), 0.4 ml of isopropanol/H_2O (volume ratio, 3:2) mixture and 32 μl Nafion solution (5 wt%) were mixed and then ultrasonicated for about 30 min to form a homogeneous ink. After that, a certain volume of dispersion was dropped onto the glassy carbon electrode and then dried at room temperature. The average loading catalyst was ~0.05 mg_{Pt} cm^{-2}.

Based on HCP202 carbon paper, 5 mg of each catalyst and electric conduction with 100 μl of 5 wt% Nafion solution (Sigma-Aldrich) was dispersed in 1 ml a water/isopropanol mixed solvent (3:1 volume ratio) by 30 min of sonication to form a homogeneous ink. Then 50 microliters of the solution were dropped onto the surface of carbon paper (1 cm^2), with a mass density of 0.2 $mg_{PBN-300-Ir}$ / cm^2, and then the sample was dried at room temperature.

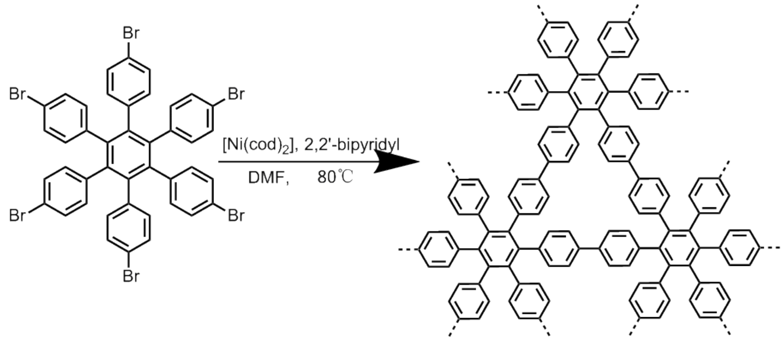

S1-2: Synthesis of PHN [S2, S3]

1,5-cyclooctadiene (cod, 1.05 mL, 8.32 mmol, Aldrich, dried over CaH_2) was added to a solution of bis(1,5-cyclooctadiene)nickel(0) ([Ni(cod)$_2$], 2.25 g, 8.18 mmol) and 2,2'-bipyridyl (1.28 g, 8.18 mmol) in dehydrated DMF (120 mL), and the mixture was heated at 80 ˚C for 1 h. To the resulting purple solution was added Hexakis(4-bromophenyl)benzene (1.007 g, 1 mmol) at 80°C, and the mixture was stirred at the temperature overnight to obtain a deep purple suspension. After cooling to room temperature, concentrated HCl was added to the mixture. After filtration, the residue was washed with $CHCl_3$ (5×30 mL), THF (5×30 mL) and H_2O (5×30 mL), respectively, and dried in vacuo to give PHN as an off-white powder (452 mg, 85.12% yield).

S1-3: Synthesis of PBN [S4]

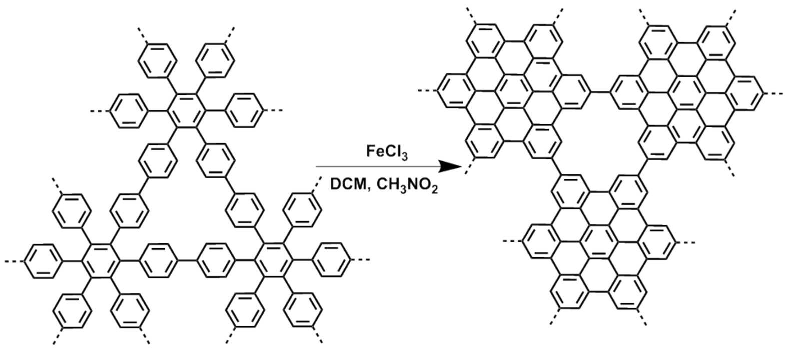

Scheme S1. Preparation scheme for PBN

To a suspension of PHN (265 mg) in dichloroform (300 mL) was added a solution of $FeCl_3$ (7.238 g, 45 mmol). After 24 hours, the precipitate was filtered off, washed by copies of dichloroform and then CH_3OH. After washing, the solid was sonicated in 100 ml water for 30 minutes. After filtration, the obtained solid was dried under reduced pressure to give a dark brown solid (PBN, 242 mg, 93% yield).

S1-4: Incorporation of Other Elements

Typical procedure: A 40 ml Telfon reactor with10 mg PBN and 1 mg $Ir(CO)_3Cl$ powder was filled with argon gas, and then sealed and heated in an oven with the heating rate of 10^oC/min up to 300 ^oC, at which temperature the reactor was heated for another 450 min. The sample was obtained after cooling.

The synthesis of PBN-300-M (M= Fe, Ni, Co, Mn, Pd, Mo, W, Re, Ir) were the same to that of PBN-300-Ir with the substitution of $Ir(CO)_3Cl$ powder by $Fe(C_5H_5)_2$, $Co(C_5H_5)_2$, $Ni(COD)_2$, $Pd(P(t\text{-}Bu)_3)_2$, $Mn(CO)_5Br$, $Mo(CO)_6$, $W(CO)_6$, $Re(CO)_5Cl$ respectively.

PBN-100-Ir，PBN-200-Ir，PBN-300-Ir，PBN-400-Ir and PBN-500-Ir was obtained when PBN: Ir was 10mg:1mg and calcined at different temperatures for 6 hours。

PBN-300-$Ir_{0.8}$，PBN-300-Ir and PBN-300-$Ir_{1.4}$ were obtained by calcination at 300 ℃ for 6 hours using $Ir(CO)_3Cl$ 0.8mg, 1mg, 1.4mg per 10 mg PBN, respectively.

S1-5: Computational Methods

The reported DFT calculations of vibrational spectra have been carried out with CRYSTAL14 at the B3LYP and TZVP basis set level on the corresponding equilibrium geometries, which was known to provide a reliable description of Raman and IR spectra[S5, S6].

The calculation of PBN dispersed in water and Gibbs free energy of hydrogen adsorption were performed using the Vienna Ab initio Simulation Package (VASP)[S7, S8] code with a plane-wave basis set. The exchange–correlation potential is the Perdew–Burke–Ernzerhof (PBE) generalized gradient approximation (GGA) functional[S9].The ion-electron interaction was described by the projected augmented wave (PAW) potential[S10]. The cut off energy for the plane-wave base was set to 500 eV.

The vacuum layer was set to 15 Å and the k-space was sampled with a grid of 3 × 3 × 1 under the Monkhorst−Pack scheme. The convergence criterion of 10^{-6} eV for electron energy and convergence criterion of 10^{-2} eV/Å for the forces on each ion were used to optimize the structures. The van der Waals interaction was considered using the DFT-D2 scheme[S12]. Charge analysis was based on the population of the Bader charge scheme[S13]. Crystal orbital Hamilton population (COHP) was calculated by Lobster[S14,S15].

The performance of the catalyst was evaluated by $\triangle G_{H*}$ (* + H+ + e− → H*) where * is the surface of the catalyst. Among them, $\triangle G_{H*}$ is obtained by calculating the total energy of adsorbed hydrogen, and then the zero point energy and entropy contribution at room temperature are corrected, expressed by $\triangle G_{H*} = \Delta E + \Delta ZPE - \Delta ST$[S16,S17]. ΔE_H = E(catalyst + H) − E(catalyst) − $E(H_2)/2$. E(catalyst + H) is the energy of catalyst with one H adsorption. E(catalyst) and $E(H_2)/2$ are the energies of pure catalyst and a half H_2, respectively. ΔS_H is entropy difference, ΔZPE is zero-point energy difference.

S2 Figures and Tables

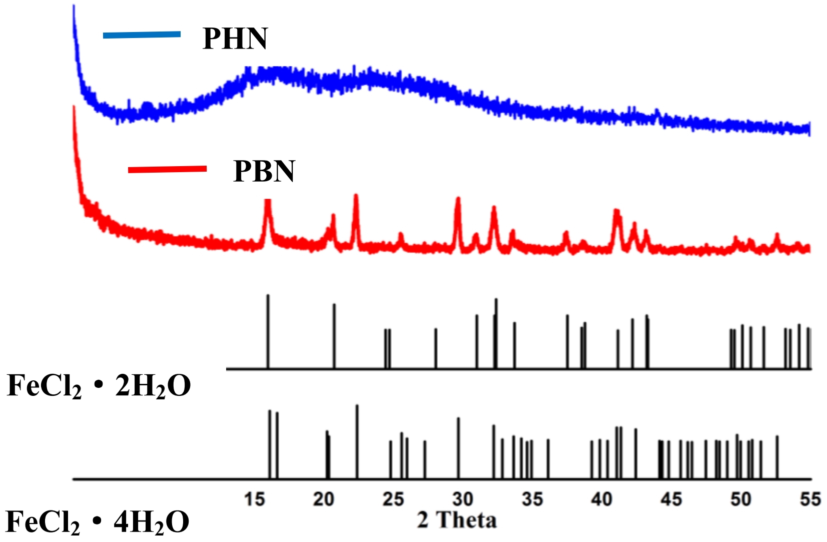

Figure S1. Powder X-ray diffraction results for PHN and PBN (unwashed) indicating the formation of iron (II) species ($FeCl_2 \cdot 2H_2O$ phase) and thus the oxidation process.

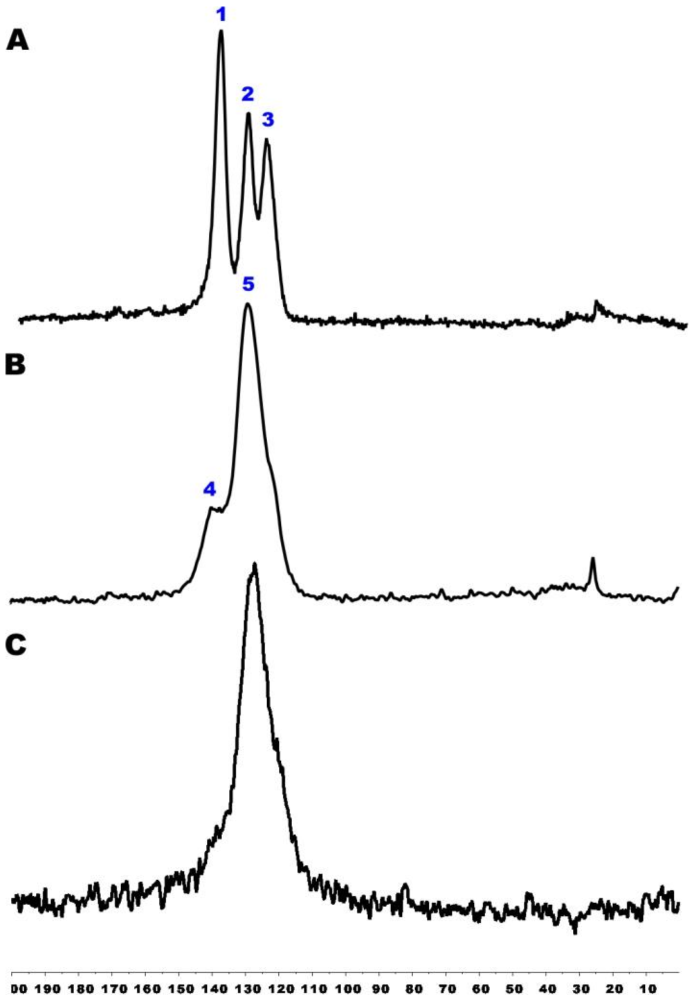

Figure S2. Solid state ^{13}C-NMR spectrum: A) is the solid state ^{13}C-NMR spectrum of PHN, the peak area ratio of 1:2:3 is 3:2.15:2.06; B) is the solid state ^{13}C-NMR spectrum of PBN, the peak area ratio of 4:5 is 1:6.2 and C) is the solid state ^{13}C-NMR spectrum of PBN-300 in which only one C peak was left.

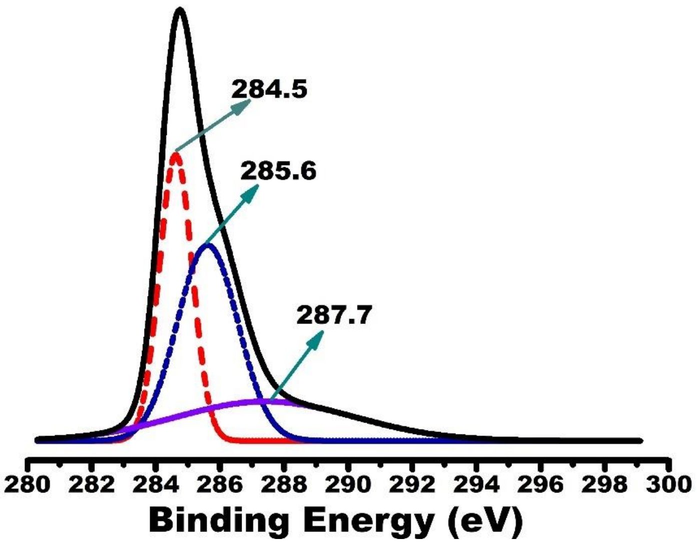

Figure S3.C 1S XPS spectrum for PHN.

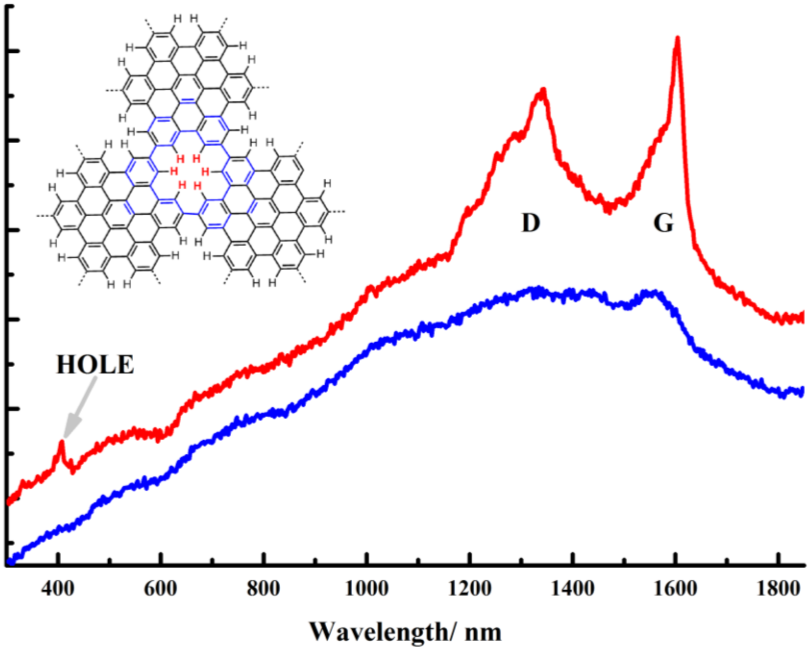

Figure S4. Raman spectrum for PHN (blue) and PBN (red).

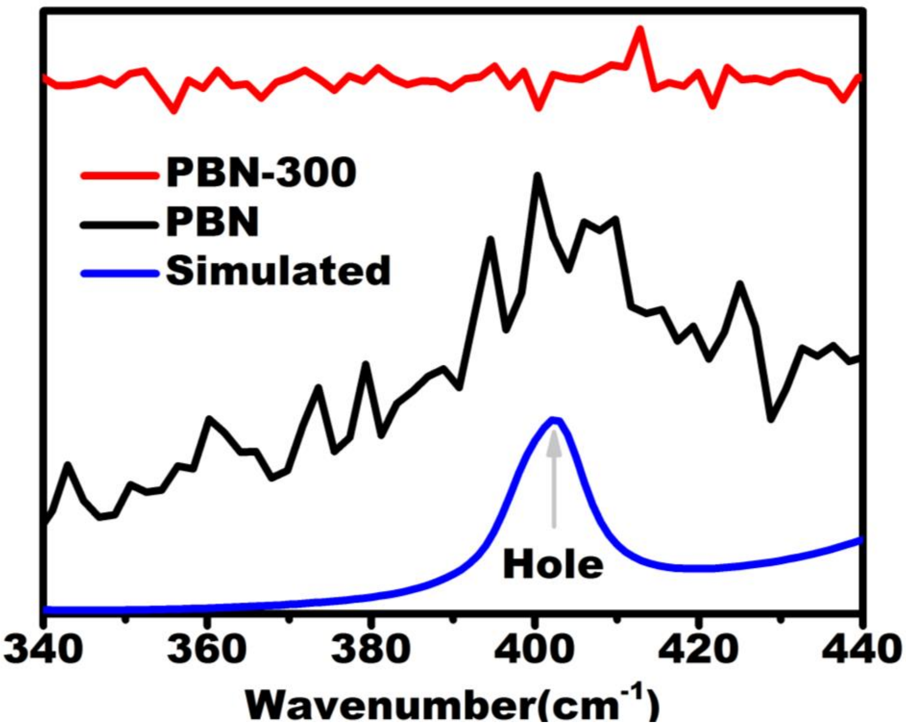

Figure S5. The comparison of Raman spectra among PBN-300, PBN and PBN (simulated). The complete disappearance of hole marker peak at around 400 cm^{-1} in PBN-300 indicated the loss of the holes after thermal treatment.

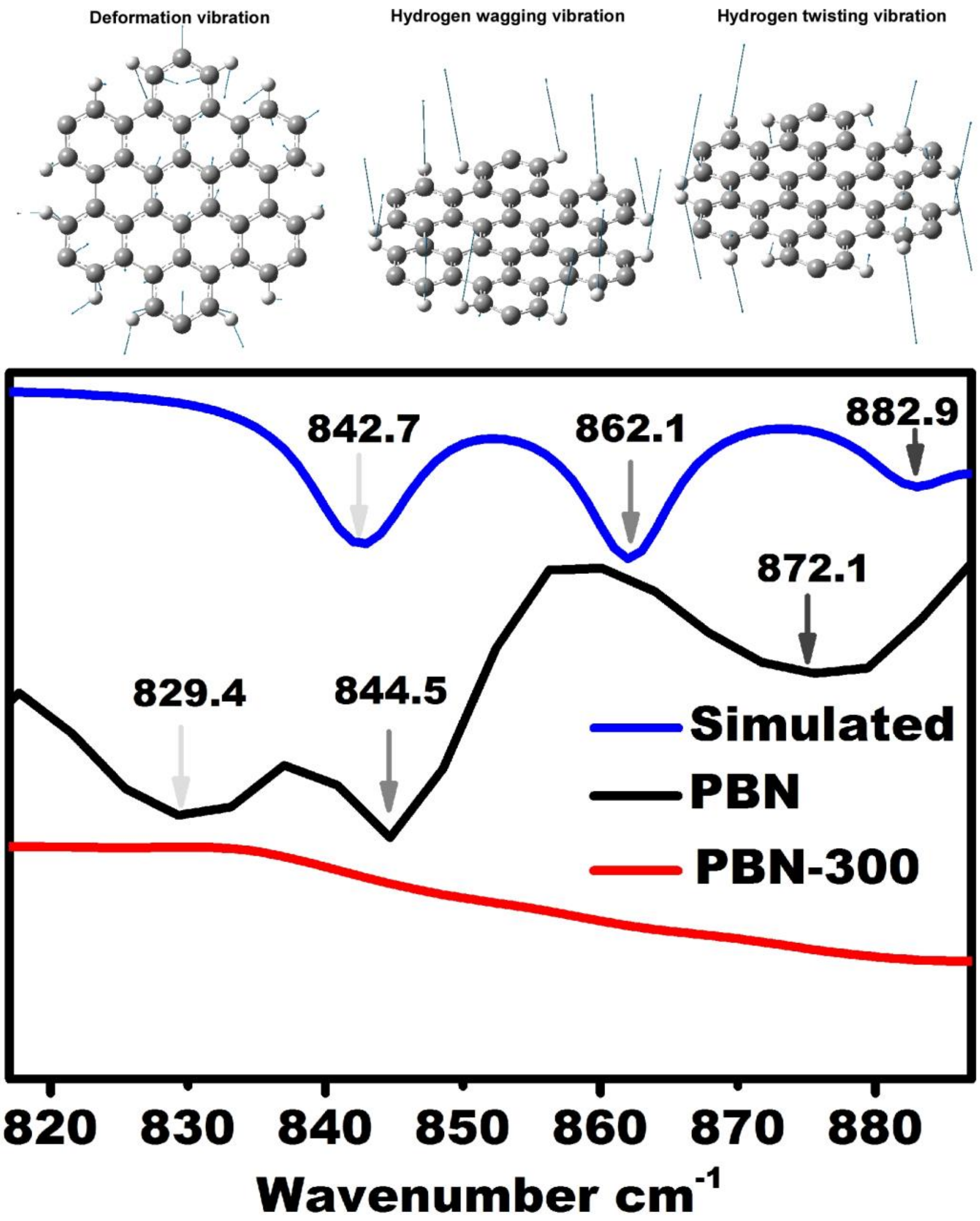

Figure S6. The comparison of IR spectra between PBN (simulated), PBN and PBN-300. The peaks of simulated PBN at 842.7, 862.1 and 882.9 cm^{-1} represented deformation vibration of hole and the hydrogen wagging (in the same direction) vibration and twisting (in the different direction) vibration of hole, respectively. The calculated IR peaks were not corrected. The complete disappearance of hole marker peak in PBN-300 indicated the loss of the holes after aging.

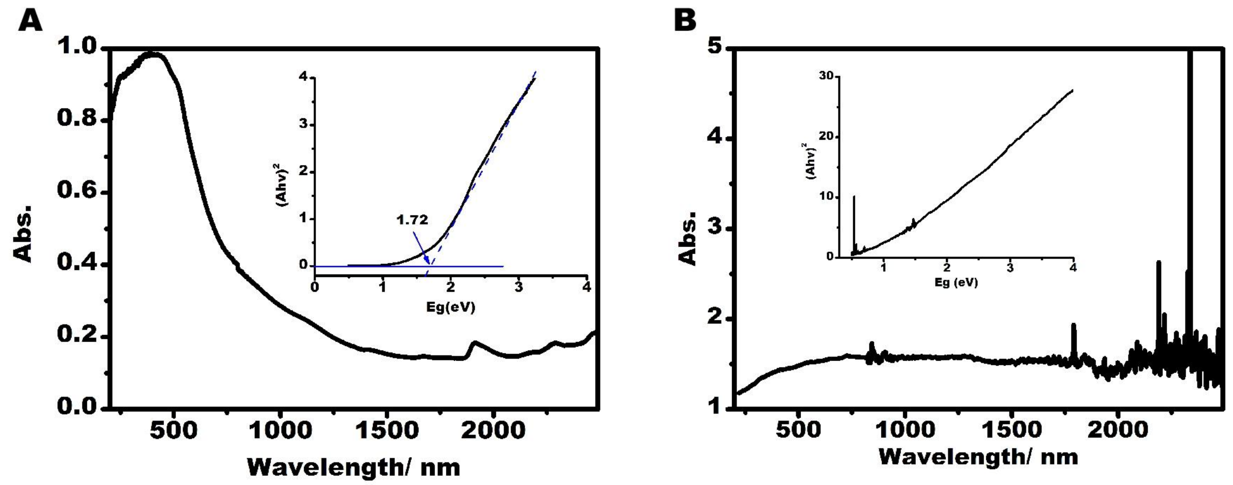

Figure S8. The comparison of band gap values between PBN powder (A) and PBN-300 (B). The band gap was around 1.72 eV for PBN, while disappeared after 300 °C treatment indicating full conjugation with the formation of graphitic carbon material. The phenomenon reflected the known instability of small holes in graphene. The measurement of the band gap was based on previous methods[S18,S19].

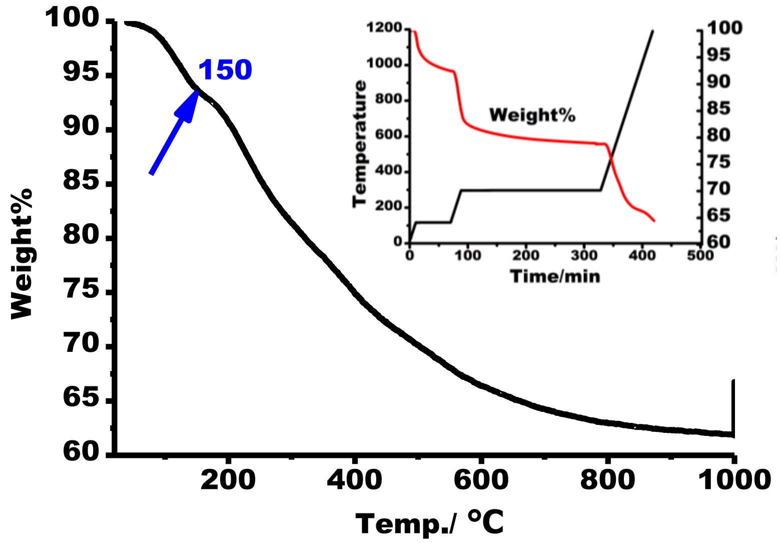

Figure S9. TGA and DSC of PBN. The 7.5% of PBN weight loss before 150 °C was due to the loss of H_2O. The weight loss (29.32%) between 150 °C and 800 °C was assigned to the loss of six CH groups per hexabenzocoronene subunit. A thermogravimetric analyzer coupled with a mass spectrometer was used to investigate the thermo-decomposition process of PBN temperature programmed TGA analysis (inset). The detailed temperature program was: first about 10 mg sample was put in a platinum crucible and heated from RT to 150 °C at a rate of 10 °C/min under N_2 atmosphere and held for 60 min; then heated up to 300 °C at a rate of 10 °C/min and held for 240 min; finally heated up to 1200 °C at a rate of 10 °C/min (the red line: weight loss rate; the black line: temperature programmed process).

TGA analysis (Figure S9) up to 800 ℃ in the nitrogen atmosphere showed that the weight loss corresponded to the releasing of around two water and six CH groups per hexabenzocorene subunit (weight loss of 34.63wt% calculated and 36.82 wt% experimental). Further analysis of the volatiles by TGA-GCMS revealed the formation of acetylene (C_2H_2), benzene (C_6H_6) and other C_nH_n derivatives, and such detected CnHn compounds strongly suggested the loss of CH groups during heating (Figure S10). Raman (Figure S5) and IR (Figure S6) analysis of PBN-300 showed the complete loss of the hole marker peaks mentioned above, also indicative the vanishing of these sub-nanometer holes. ^{13}C-NMR (Figure S2) analysis showed a single peak, indicating the carbons in PBN-300 are indistinguishable due to the loss of CH groups. TEM imaging of PBN-300 (Figure S11) revealed the perseverance of the sheet-like morphology but apparent loss of the hole features.

We speculated that the loss of CH groups would lead to momentary formation of carbon defects, which then self-healed to form a conjugated carbon network (Figure S11).

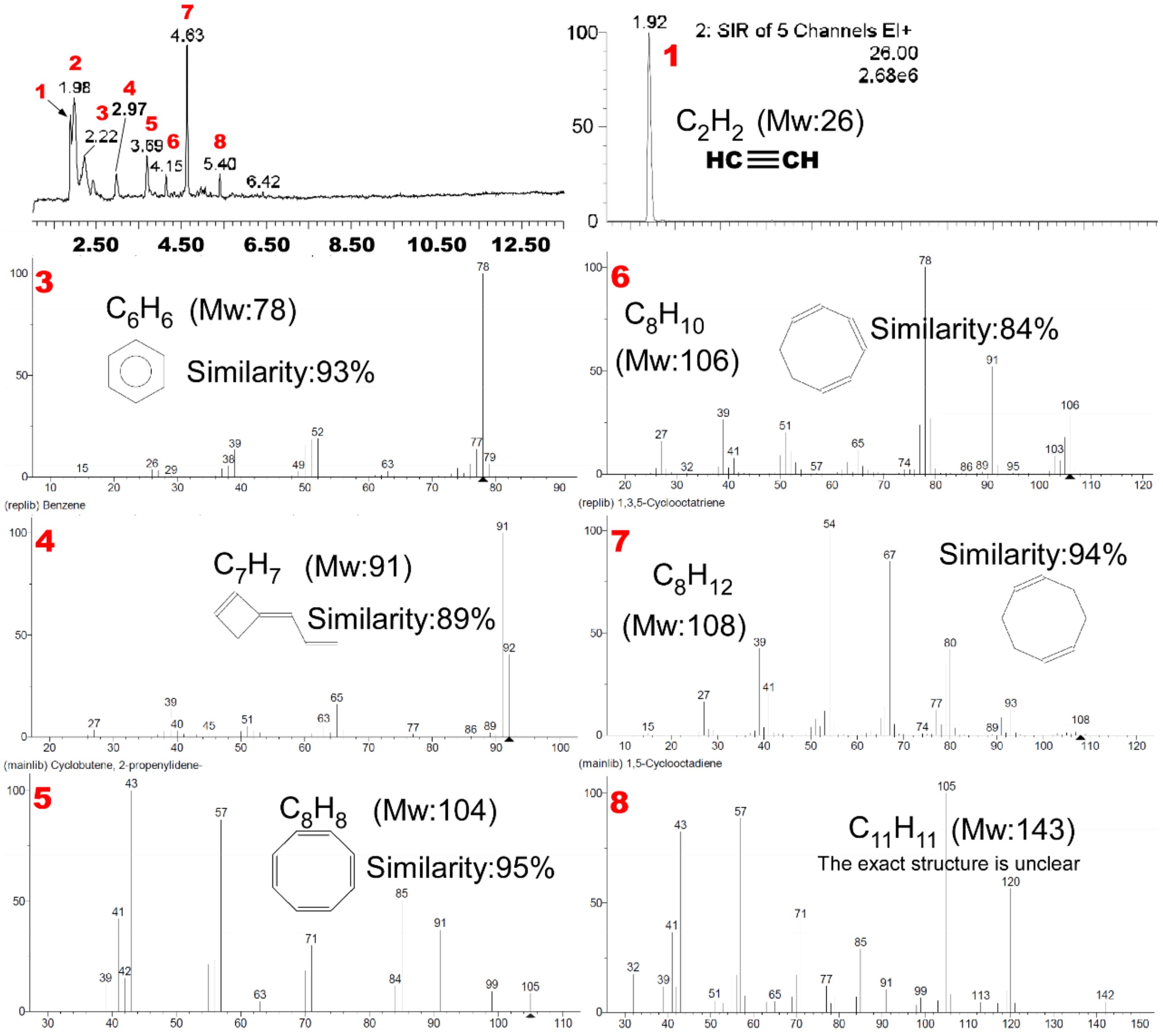

Figure S10. The TGA-GC-MS spectrum of PBN in nitrogen with a heating rate of 10 ℃/min. Peak 2 corresponded to nitrogen gas. The detection of C_2H_2 (peak 1), C_6H_6 (peak 3), C_7H_7 (peak 4), C_8H_8 (peak 5), $C_{11}H_{11}$ (peak 8), cyclooctadiene/cyclooctatriene (derivatives of C_8H_8) strongly indicated the loss of CH groups around the holes, which at the same time led to the healing process to produce conducting graphitic materials. The identity of acetylene (peak 1) was confirmed by the retention time comparison with that of pure acetylene gas.

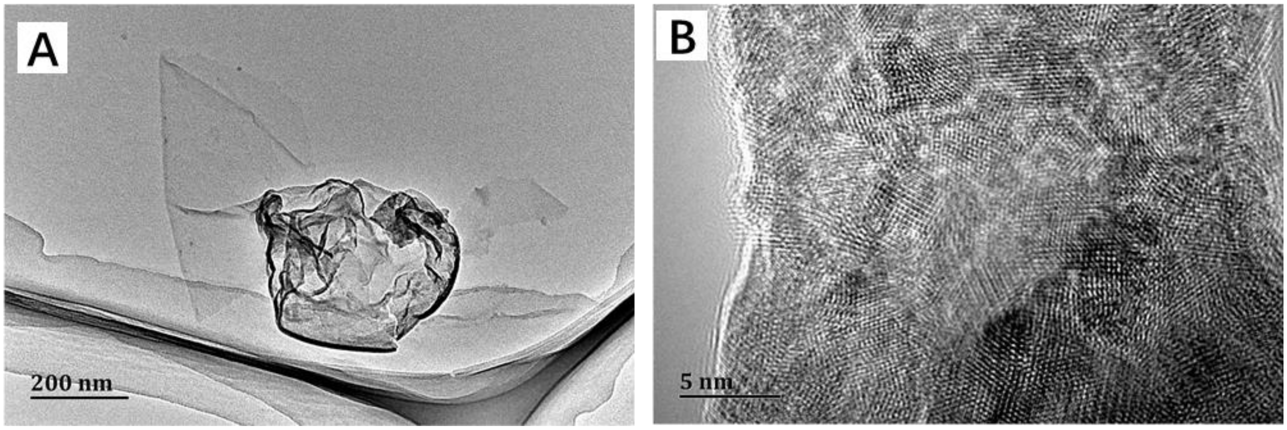

Figure S11. The TEM (A) and HR-TEM (B) images PBN-300. After high temperature treatment, the pore-like features disappeared, and the crystalline carbon material was obtained.

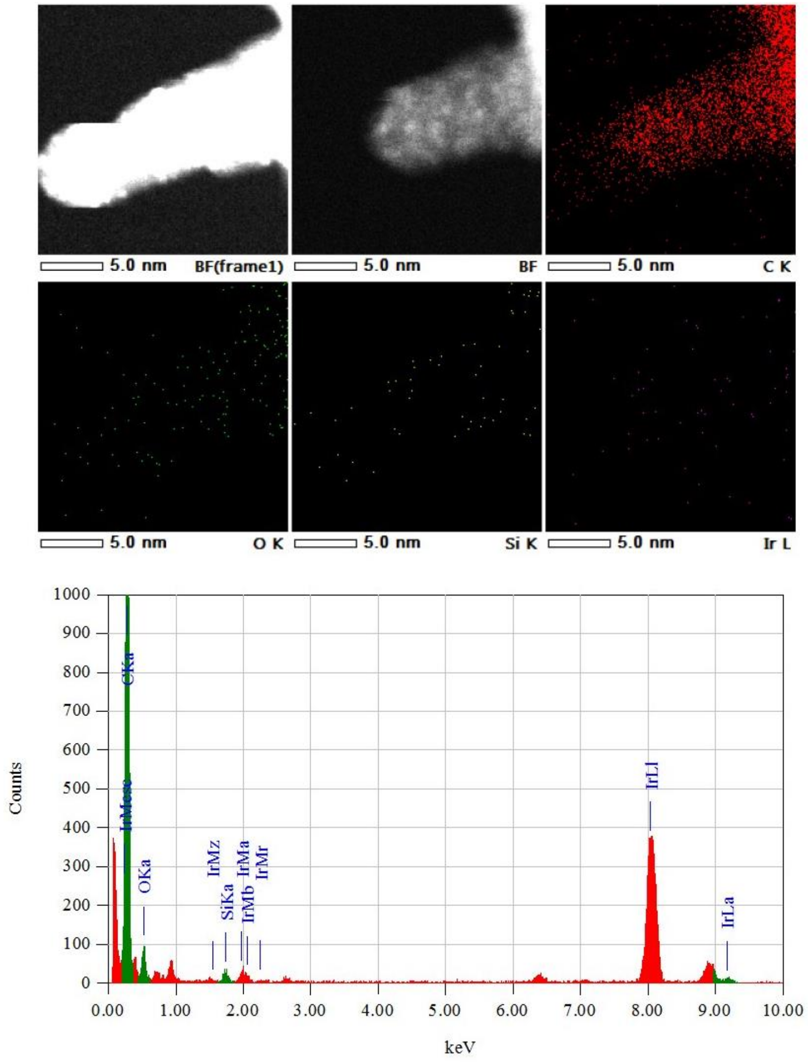

Thin Film Standardless Standardless Quantitative Analysis
Fitting Coefficient : 0.4708

Element	(keV)	Mass%	Counts	Sigma	Atom%	Compound	Mass%	Cation	K
C K (Ref.)	0.277	94.97	9246.36	0.77	97.29				1.0000
O K !	0.525	2.76	526.29	0.15	2.12				0.5110
Si K !	1.739	1.17	238.05	0.14	0.51				0.4793
Ir L	9.174	1.10	97.42	0.21	0.07				1.0961
Total		100.00			100.00				

Figure S12. The ACHAADF-STEM and corresponding EDS mapping images of PBN-300-Ir treated at 300 °C. From the standardless quantitative analysis about 1.1% wt of Ir in the PBN-300-Ir sample which was higher than the content detected by ICP-MS (0.74%). Since the ICP-OES is reflective of the bulk content, and therefore is more reliable than the EDS value.

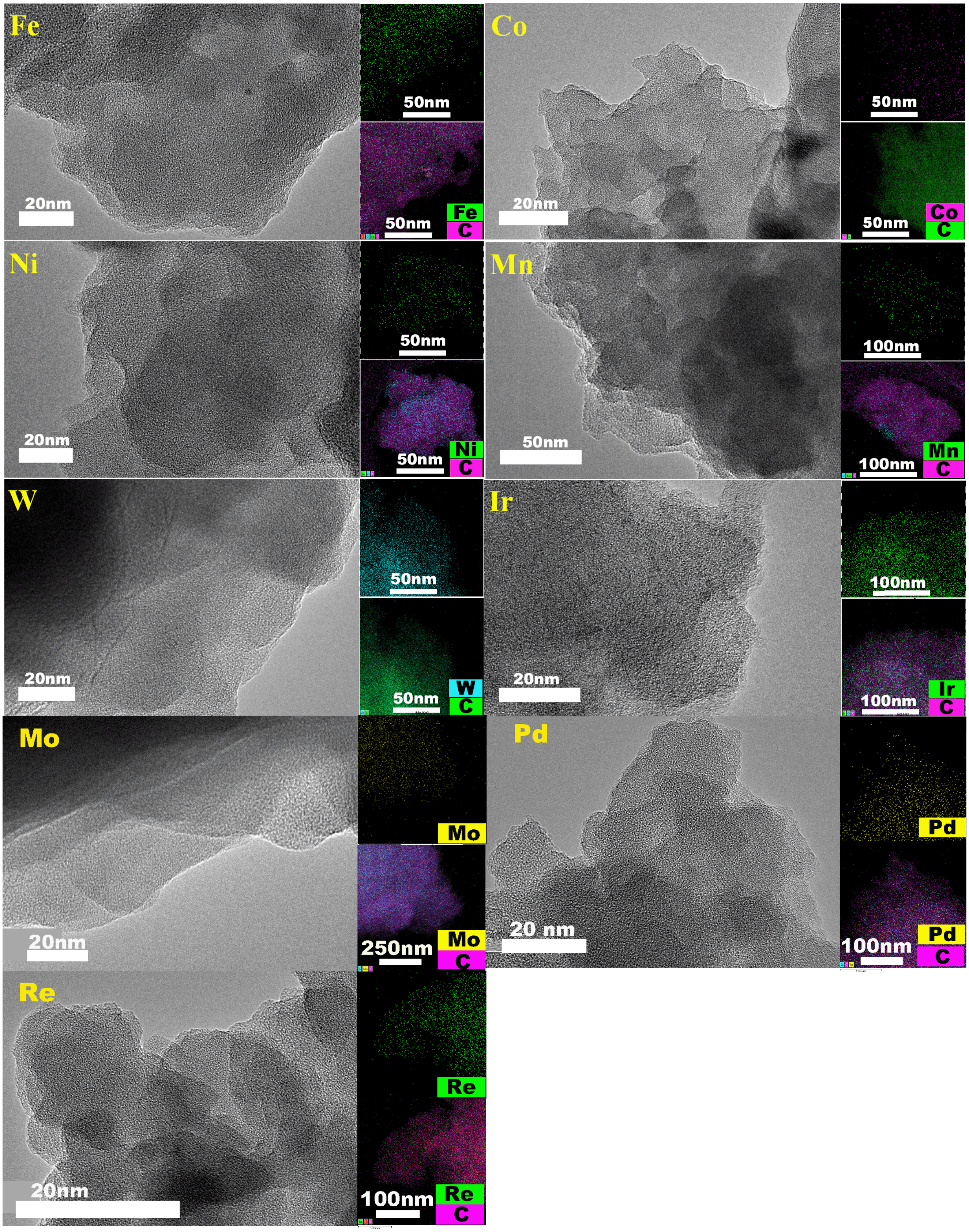

Figure S13. The HR-TEM images and corresponding EDS mapping images of PBN-300-M (M= Fe, Co, Ni, Mn, Pd, Mo, W, Re, Ir, Mo, Pd, Re).

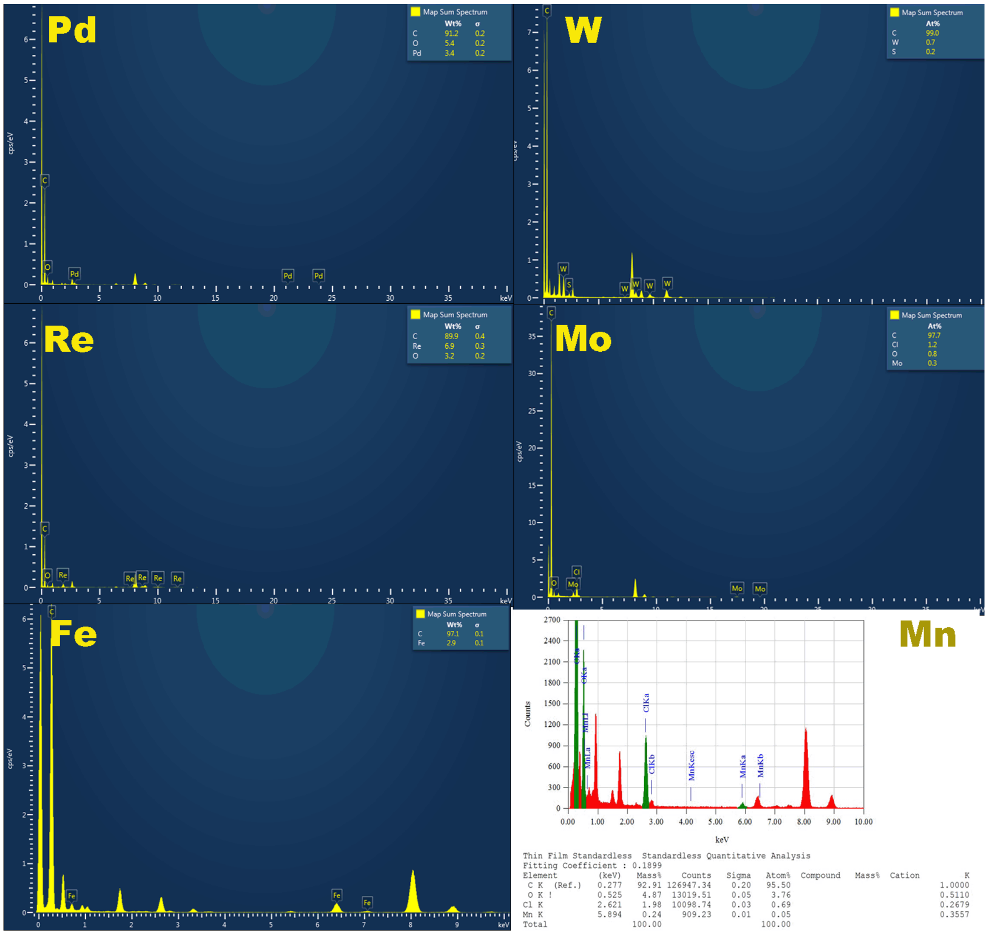

Figure S14. The EDS diagram of Pd, W, Re, Mo, Fe, Mn

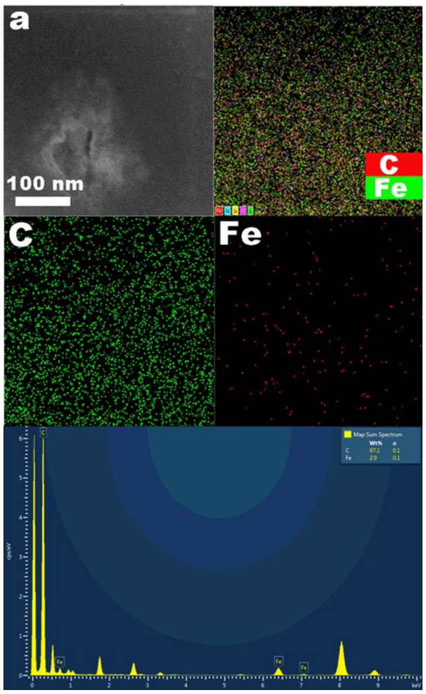

Figure S15. HRTEM mapping and EDS results of PBN-300-Fe.

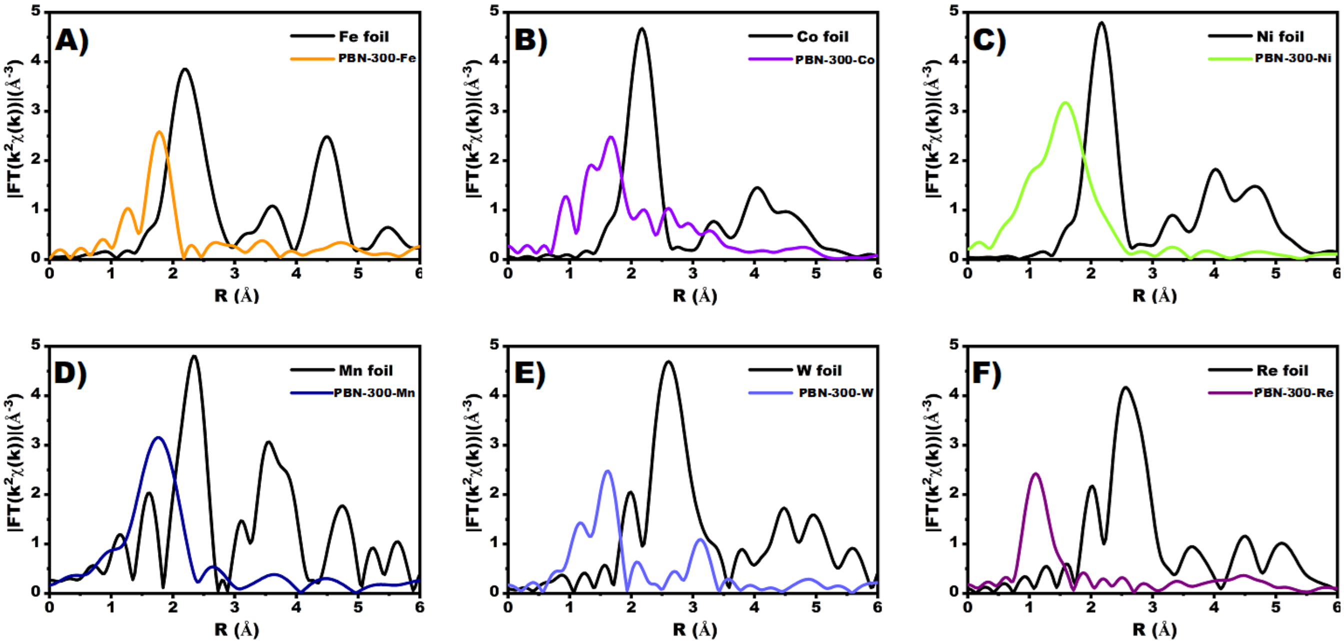

Figure S16. EXAFS data for PBN-300-M, corresponding metal foils (M= Fe, Co, Ni, Mn, W and Re respectively).

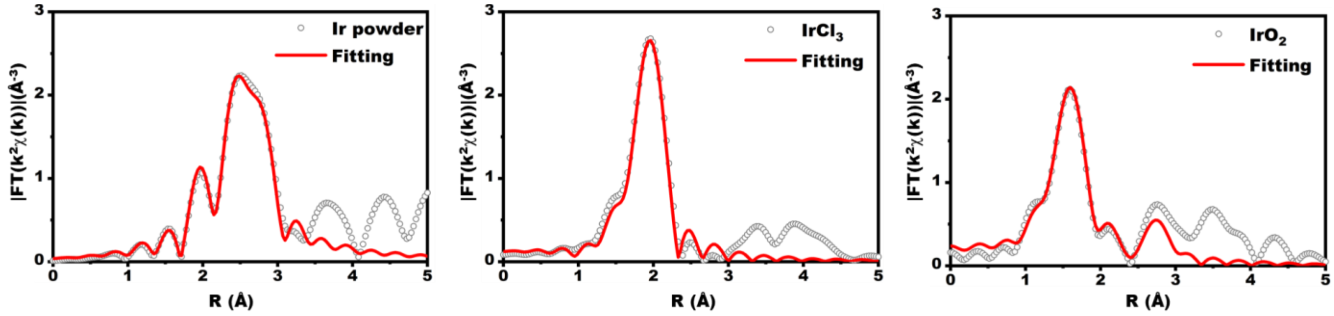

Figure S17. Quantitative EXAFS fitting curves of the reference Ir powder, $IrCl_3$ and IrO_2.

Table S1. Structural parameters of the reference Ir powder, $IrCl_3$ and IrO_2 extracted from quantitative EXAFS fitting curves. ($S0_2 = 0.74$)

Structure NO.	path	N	R (Å)	σ^2 (Å^2)	ΔE0 (eV)	R
**Ir Powder**	Ir-Ir	12	2.70698	0.00266	7.839	0.004
**$IrCl_3$**	Ir-Cl_1	4	1.97362	0.00272	8.511	0.009
	Ir-Cl_2	2	2.21603	0.00022		
	Ir-Ir	0.8	2.36320	0.01123	62.493	
**IrO_2**	Ir-O_1	4	1.88071	0.00199	9.806	0.036
	Ir-O_2	2	2.00170	0.00189		
	Ir-Ir	2	2.94854	0.00156	17.702	

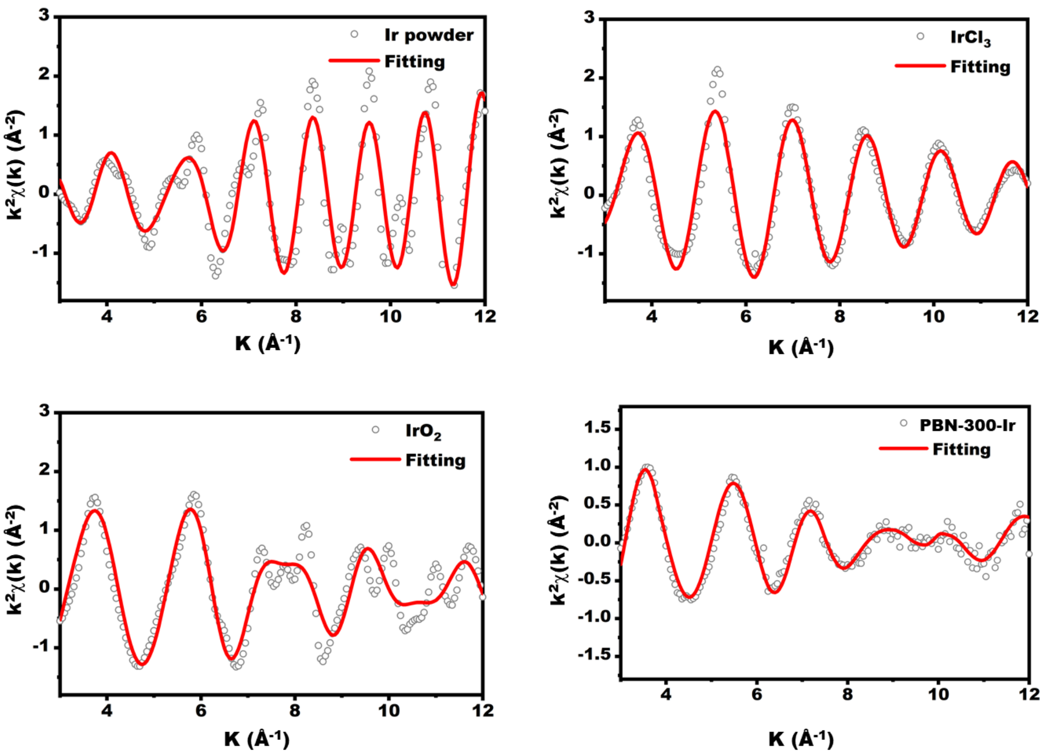

Figure S18. Ir L3-edge EXAFS of Ir powder, $IrCl_3$, IrO_2 and PBN-300-Ir in k spaces.

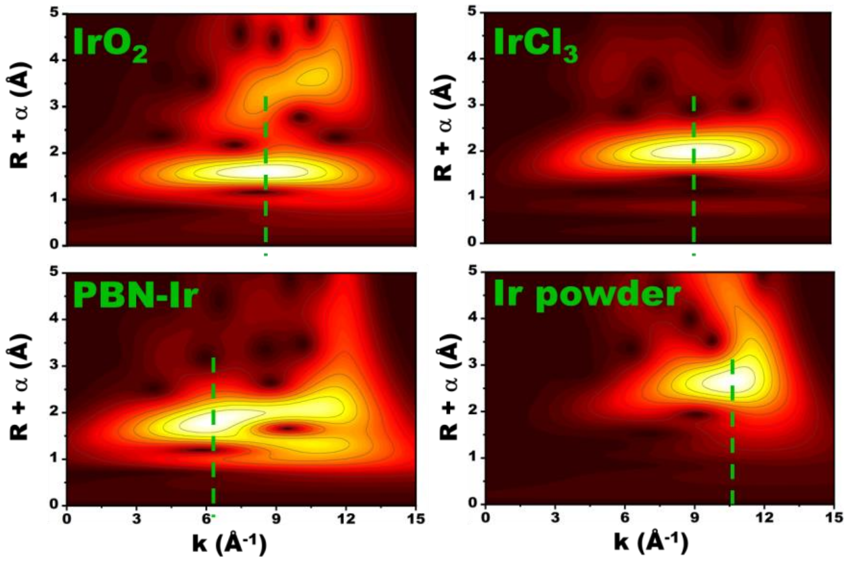

Figure S19. Wavelet transforms for the k^2-weighted Ir L3-edge EXAFS signals of IrO_2, $IrCl_3$, PBN-300-Ir. and Ir powder.

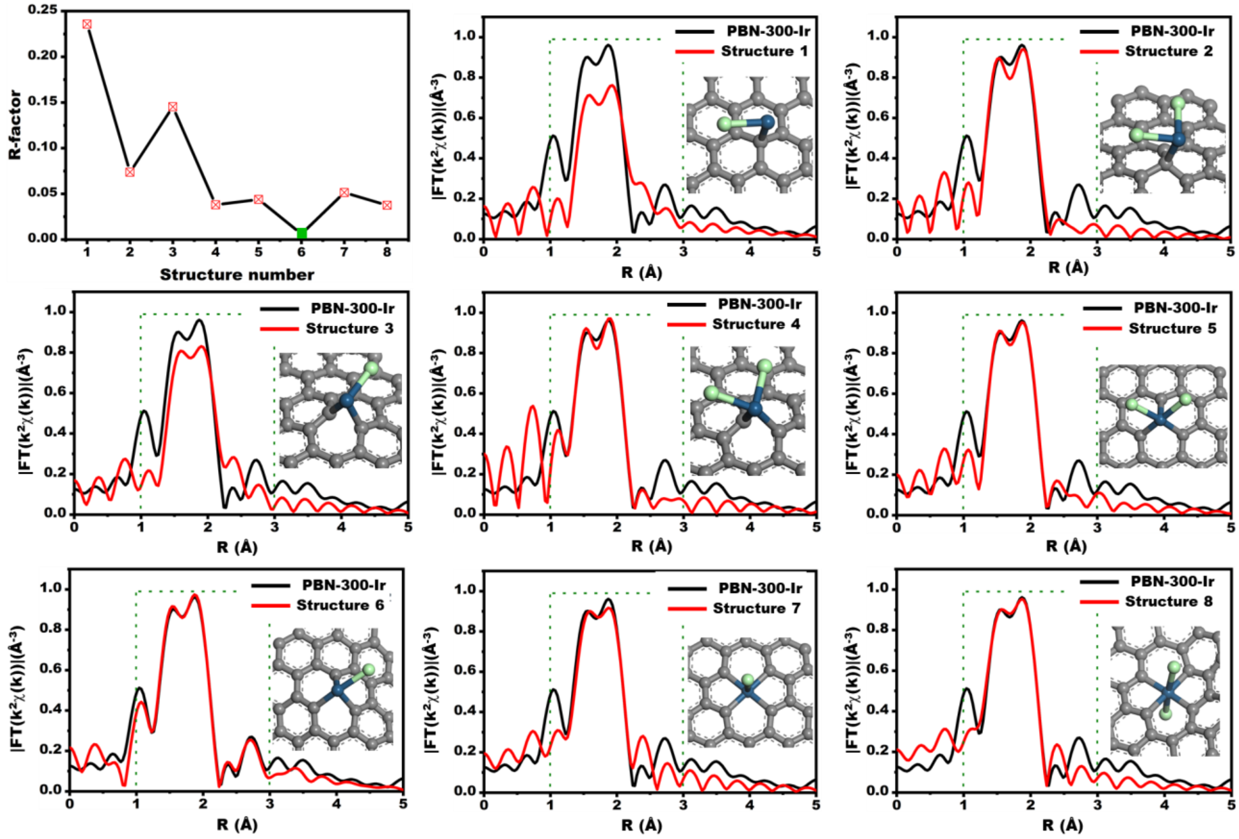

Figure S20. The R-factors plot and the corresponding structural models for the PBN-300-Ir single catalyst. The green dot represented the best model.

Table S2. Ir L3-edge EXAFS curve fitting parameters of PBN-300-Ir single atom catalyst.

Structure NO.	path	N	R (Å)	σ^2 ($Å^2$)	ΔE0 (eV)	R
**1**	Ir-C	1	2.02530	0.00928	4.610	0.236
	Ir-Cl	1	2.27240	0.00524	-0.496	
**2**	Ir-C	1	1.97362	0.01217	3.488	0.074
	Ir-Cl_1	1	2.21603	0.01061	0.696	
	Ir-Cl_2	1	2.36320	0.00904	0.696	
**3**	Ir-C	2	2.05218	0.00593	6.782	0.145
	Ir-Cl	1	2.29190	0.00513	0.137	
**4**	Ir-C_1	1	1.86513	0.00930	-0.407	0.038
	Ir-C_2	1	1.97426	0.01588		
	Ir-Cl_1	1	2.21335	0.01340	-1.637	
	Ir-Cl_2	1	2.36345	0.01146		
**5**	Ir-C_1	2	2.02593	0.00901	9.072	0.044
	Ir-C_2	1	2.15007	0.01306		
	Ir-Cl	2	2.34197	0.00511	2.717	
**6**	Ir-C_1	1.9	1.97680	0.001744	8.181	**0.006**
	Ir-C_2	0.9	2.07320	0.002112		
	Ir-C_3	0.7	2.39990	0.001981		
	Ir-Cl	1	2.40990	0.00520	1.346	
**7**	Ir-C	4	2.07418	0.00081	9.123	0.051
	Ir-Cl	1	2.30714	0.00360	0.169	
**8**	Ir-C	4	2.04516	0.00068	7.384	0.038
	Ir-Cl	2	2.29223	0.00075	-1.733	

N, coordination number; R, distance between absorber and backscatter atoms; σ^2, Debye-Waller factor to account for both thermal and structural disorders; ΔE_0, inner potential correction; R factor indicates the goodness of the fit. Error bounds (accuracies) that characterize the structural parameters obtained by EXAFS spectroscopy are estimated as N ± 10%; R ± 1%; σ^2 ± 20%; ΔE_0 ± 20%. $S0_2$ was fixed to 0.74 as determined from Ir powder fitting. Coordination number (N) fixed according to the crystal structure. Fitting range: 3.0 ≤ k (/Å) ≤ 12 and 12 ≤ R (Å) ≤ 3. The path parameters ΔE_0 and σ^2 of the same atoms were set uniform. The R-factor should be stated that: R-factor<0.02 means the model is good, 0.02<R-factor<0.05 means the models slightly deviate or data quality is not good enough, 0.05<R-factor<0.1 means the models greatly deviate or data quality is bad, R-factor >0.1 means the models is wrong. All the fitting variables using custom guess method.

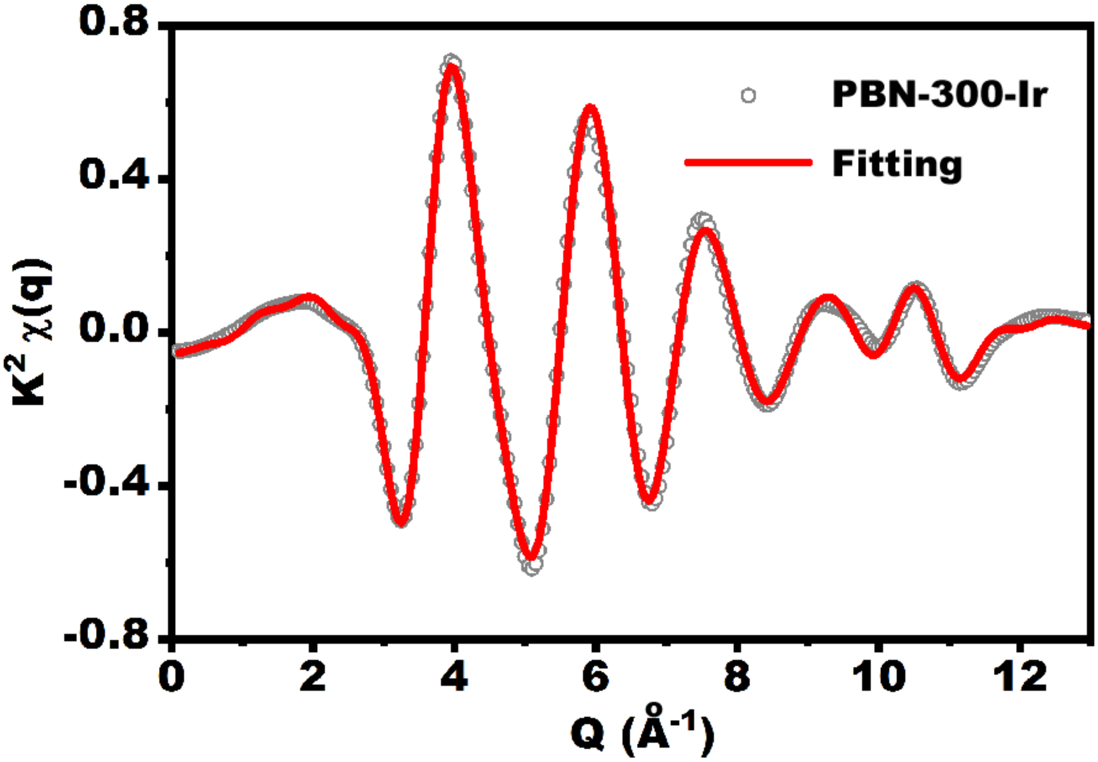

Figure S21. EXAFS spectrum fitting curves of **PBN-300-Ir** at Q space.

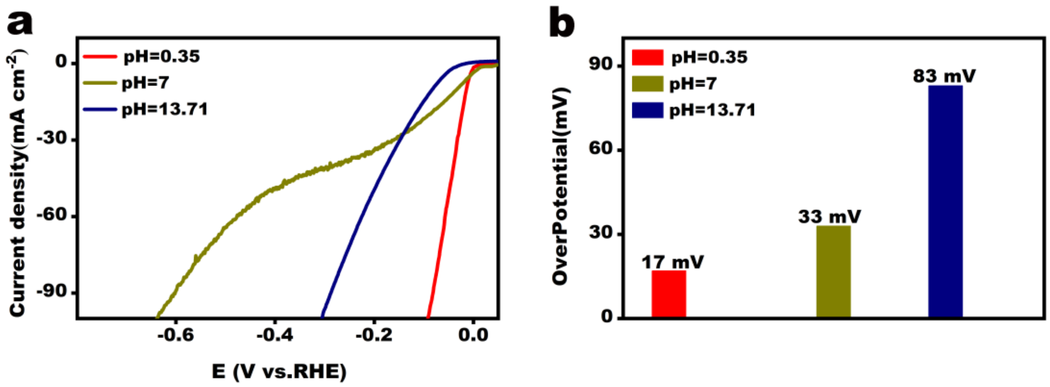

Figure S22. PBN-300-Ir as HER catalyst under different pH values (pH=0.35 H_2SO_4 , pH=7 0.1M PBS and pH=13.71 1M KOH).

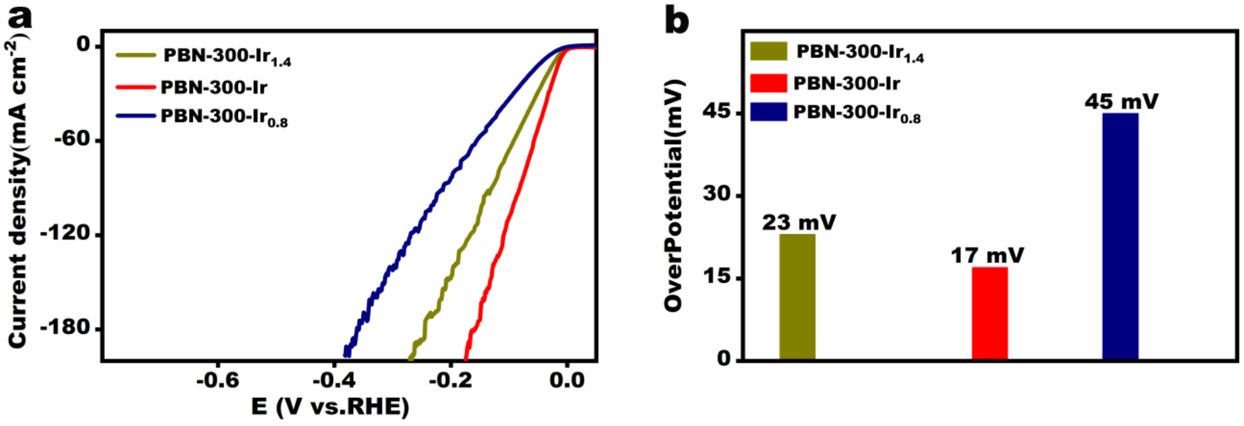

Figure S23. The effect of different Ir precursor content on HER activity of PBN-300-Ir was in 0.5 M H_2SO_4

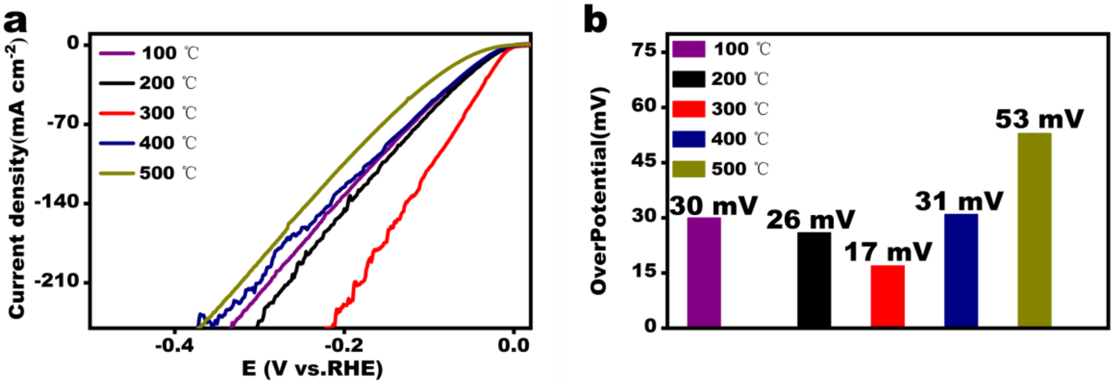

Figure S24. The effect of different calcination temperatures on the HER activity of PBN-T-Ir in 0.5 M H_2SO_4, where T stands for the calcination temperature.

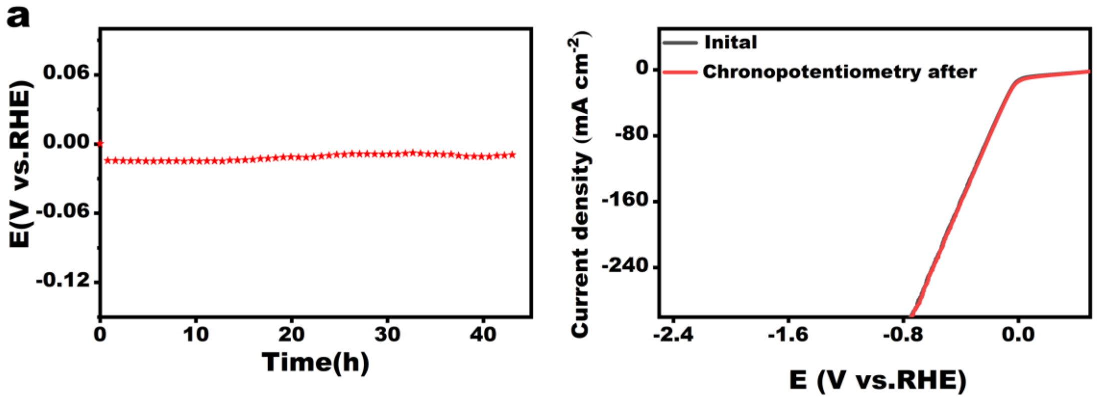

Figure S25. Chronopotentiometry of PBN-300-Ir under constant current density without iR correction.

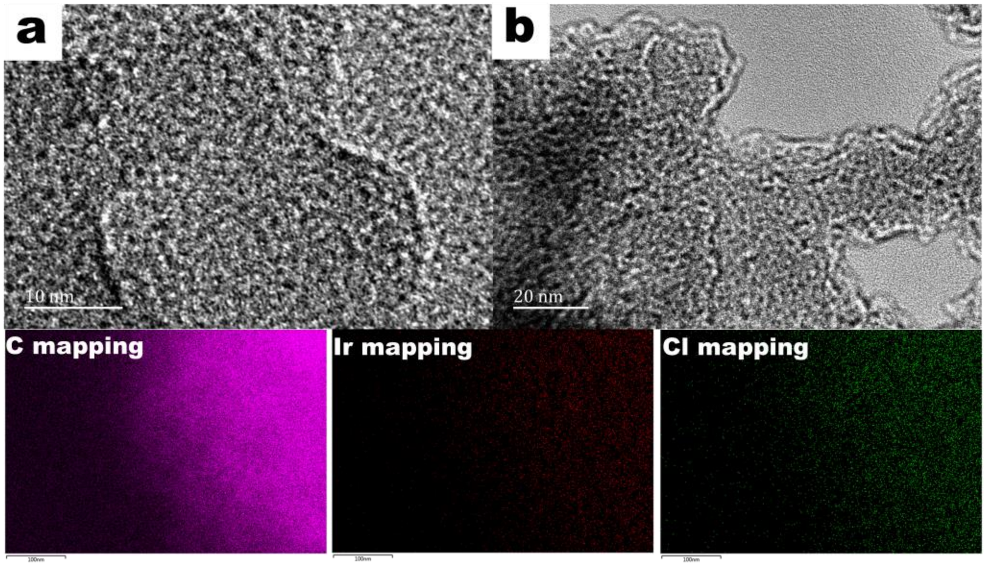

Figure S26. HRTEM diagram after PBN-300-Ir test Chronoamperomet stability a) 10 nm b) 20 nm and the mapping of C, Ir and Cl.

Table S3. Comparison of HER performance for PBN-300-Ir with other selected electrocatalysts.

**Catalysts**	**Electrolyte**	**TOF (η= 100 mV)**	**Ref.**
**PBN-300-Ir**	0.5 M H_2SO_4	41.29	**This work**
Ir/C	0.5 M H_2SO_4	2.30	**This work**
Pt/C	0.5 M H_2SO_4	1.37	**This work**
Pt/OLC	0.5 M H_2SO_4	40.78	S20
Pt/CoSe	1.0 M PBS	3.93	S21
Pt@PCM	0.5 M H_2SO_4	10(200mV)	S22
PtRu/RFCS	0.5 M H_2SO_4	0.375	S23
Rh1-TiC	0.5 M H_2SO_4	5.968(25 mV)	S24

Table S4. Adsorption free energies of H (△G_H eV) on supported on the C_xN_yIrCl-H.

**samples**	C_2NIrCl	CN_2IrCl	N_3IrCl
**△G_H (eV)**	-0.107	-0.837	-1.001

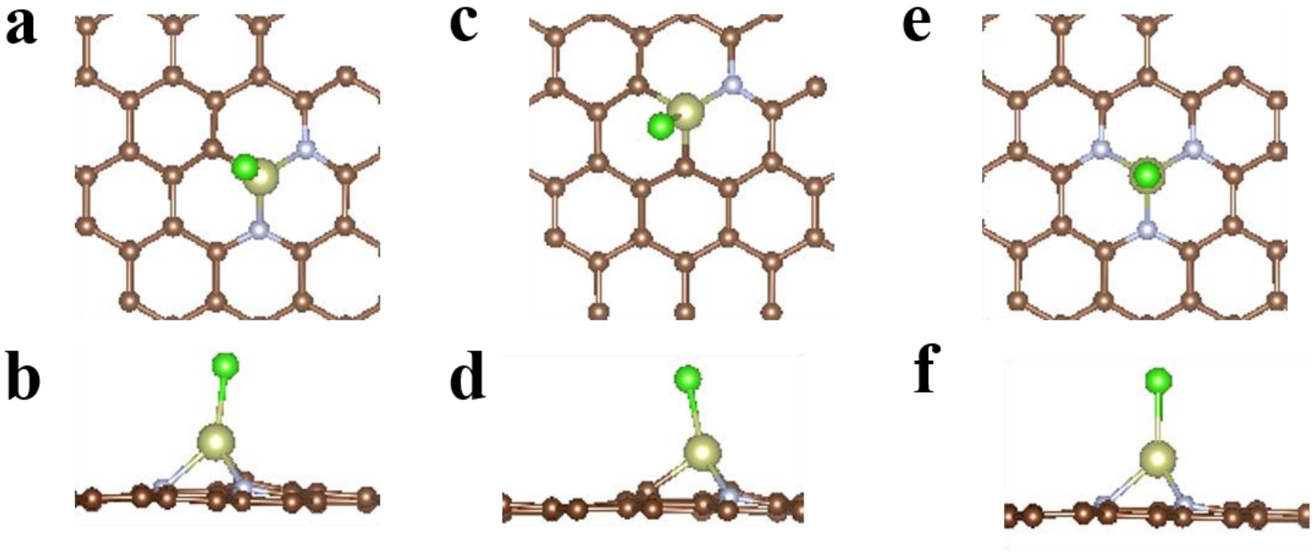

Figure S27. a) Top and b) side view of optimized CN_2IrCl structure. c) Top and d) side view of optimized C_2NIrCl structure. e) Top and f) side view of optimized N_3IrCl structure. g) Top and h) side view of optimized C_3NIrCl structure. i) Top and j) side view of optimized C_2N_2IrCl structure. k) Top and l) side view of optimized CN_2IrCl structure.

S3 Modeling

S3-1: Structural file for the simulation of electron diffraction pattern

The off-set stacking model of PBN was constructed with the space group of P63/MMC in hexagonal lattice with the cell parameter of a = b = 12.88 Å and c = 7.04 Å. The atomic position information is as the following:

```
_atom_site_label
_atom_site_type_symbol
_atom_site_fract_x
_atom_site_fract_y
_atom_site_fract_z
_atom_site_U_iso_or_equiv
_atom_site_adp_type
_atom_site_occupancy

C1   C   0.55511  -0.33333   0.25000   0.00000  Uiso   1.00

C2   C   0.77534  -0.33333   0.25000   0.00000  Uiso   1.00
```

C3 C 1.22341 -0.33333 0.25000 0.00000 Uiso 1.00

C4 C 0.77284 -0.22653 0.25000 0.00000 Uiso 1.00

C5 C 0.66561 -0.22366 0.25000 0.00000 Uiso 1.00

H6 H 0.85708 -0.14230 0.25000 0.00000 Uiso 1.00

C7 C 0.89382 0.55986 0.25000 0.00000 Uiso 1.00

C8 C 1.00106 0.55699 0.25000 0.00000 Uiso 1.00

H9 H 0.80959 0.47563 0.25000 0.00000 Uiso 1.00

S3-2: Structural file for the simulation of vibrational spectra

The atomic position information is as the following:

_symmetry_space_group_name_H-M 'P 1'

_cell_length_a 12.8311641

_cell_length_b 12.8310656

_cell_length_c 20

_cell_angle_alpha 90

_cell_angle_beta 90

_cell_angle_gamma 119.999732

loop_

_atom_site_label

_atom_site_type_symbol

_atom_site_fract_x

_atom_site_fract_y

_atom_site_fract_z

H1	H	0.189280480898	-0.478322147402	0.514162282945
H2	H	-0.189281160444	0.478320887715	0.514156725877
H3	H	0.332386060204	-0.189300973980	0.514073103849
H4	H	0.478328133501	-0.332376246138	0.514139000396
H5	H	-0.478327883380	0.332375970737	0.514135821185
H6	H	-0.332386218862	0.189302639414	0.514061863394
H7	H	-0.189279755512	0.332398538545	0.485837071797
H8	H	-0.332385606045	0.478310984665	0.485939381571
H9	H	-0.478327613069	0.189296073373	0.485865032728
H10	H	0.478328074856	-0.189295650343	0.485859168166
H11	H	0.332385376747	-0.478312239523	0.485932071469
H12	H	0.189280624539	-0.332397267510	0.485840009139
C1	C	-0.000000061476	0.109977836662	0.499999032791
C2	C	-0.000000017430	-0.109977988635	0.500001797950
C3	C	0.109976213904	-0.000000838883	0.499998595516
C4	C	-0.109976272596	-0.109977072017	0.500001788845
C5	C	0.109976179283	0.109976916142	0.500001816869
C6	C	-0.109976308467	0.000000690048	0.499996983494
C7	C	-0.000000064670	0.221800528932	0.499999919130
C8	C	0.000000045390	-0.221800598341	0.500001546933
C9	C	0.221797304815	-0.000001331335	0.499994032064
C10	C	-0.221797350976	-0.221798648149	0.500007647503

C11	C	0.221797271105	0.221798577089	0.500007168678
C12	C	-0.221797391929	0.000001279973	0.499989682382
C13	C	0.000000204193	0.442196089218	0.500000169207
C14	C	0.000000273245	-0.442195820119	0.500000400783
C15	C	0.442194760588	-0.000000845997	0.499972067079
C16	C	-0.442195039849	-0.442195523203	0.500028891511
C17	C	0.442194940516	0.442195593101	0.500027759712
C18	C	-0.442195217655	0.000000297370	0.499970033679
C19	C	0.109456687137	0.332456870485	0.500987354435
C20	C	-0.109456738475	-0.332456937342	0.500987310692
C21	C	0.222997953362	-0.109458468618	0.500954746803
C22	C	-0.332452996912	-0.222997176878	0.500979096399
C23	C	0.332452948897	0.222997220229	0.500977517932
C24	C	-0.222998032029	0.109458573672	0.500949519446
C25	C	-0.109456767805	0.223000239580	0.499010892845
C26	C	-0.222997902325	-0.332456472944	0.499049071948
C27	C	0.332452893089	0.109455721876	0.499021569776
C28	C	-0.332453059211	-0.109455776601	0.499019867062
C29	C	0.222997921926	0.332456436613	0.499047555402
C30	C	0.109456904457	-0.223000043157	0.499014798276
C31	C	0.106228171675	0.438820612176	0.505358032953
C32	C	-0.106228748588	-0.438821350769	0.505353572014
C33	C	0.332589344331	-0.106232787706	0.505306304860

C34	C	-0.438817711938	-0.332585698974	0.505352150527
C35	C	0.438817700312	0.332585746250	0.505350629167
C36	C	-0.332589547782	0.106233096699	0.505300124165
C37	C	-0.106228157914	0.332592936796	0.494643046645
C38	C	-0.332589222500	-0.438822708467	0.494700066114
C39	C	0.438817715393	0.106231889226	0.494649517868
C40	C	-0.438817400099	-0.106231684291	0.494644352216
C41	C	0.332589240088	0.438822317229	0.494695814987
C42	C	0.106228823488	-0.332592266493	0.494646220829

S3-3: Optimized structure information of PBN-300-Ir.

```
1.00000000000000
    9.9055438212924045    0.0000000000000000    0.0000000000000000
   -4.9528565397862048    8.5783252956929186    0.0000000000000000
    0.0000000000000000    0.0000000000000000   15.0000000000000000
  C    Ir   Cl
   31     1     1
Direct
  0.9998983203701073  0.0003508344486747  0.3253749573739526
  0.1658906645719966  0.0823312579875051  0.3279388470646936
  0.2498050928217523  0.9997146225285434  0.3257709792477996
  0.4163401065018206  0.0823370026212586  0.3279513996781063
  0.5003467337775476  0.0003560467241144  0.3253703671474923
  0.6664558360082253  0.0833981695734565  0.3269312287901229
  0.7498462636152254  0.9997961215951037  0.3284416222692599
  0.9168375891562448  0.0833951977433642  0.3269235002309756
  0.0004341882875778  0.2503810057622289  0.3285025116592450
  0.1658901350363848  0.3331051634780735  0.3378487170931237
  0.2473735125452592  0.2467879619798623  0.3407691045362213
  0.4092571034174455  0.3186251852919995  0.3599787710504643
  0.4993092733629325  0.2467854938972422  0.3408109982112748
  0.6671127455375157  0.3331070645005525  0.3379160444655227
  0.7498420300629576  0.2503821952867256  0.3285361705623728
  0.9168305879429113  0.3337689560860326  0.3269451365597149
  0.9998814333648340  0.4998791665997047  0.3253636028574007
```

0.1658660187806120	0.5838862480922558	0.3278169028108024
0.2473408229723404	0.5009070117600984	0.3406209799440845
0.4092254194382505	0.5910160494508929	0.3597437725495283
0.6816555037167902	0.5910039793353832	0.3598243568504174
0.7534543094723958	0.5009094485334700	0.3407138252600461
0.9179025344812928	0.5838841049971464	0.3278726268292402
0.0005199232696640	0.7504244765279893	0.3256993921476763
0.1667069195053372	0.8335202220105273	0.3234510675540321
0.2497974394529692	0.7504270999061475	0.3256599513580340
0.4163352633076585	0.8343646492315315	0.3277925795284489
0.4993171198078628	0.7528802179006036	0.3405437761690777
0.6671188742599412	0.8343381146229518	0.3377244035747324
0.7534488206506396	0.7528687208321259	0.3405948337049907
0.9179175562406598	0.8343554539678593	0.3278358642944994
0.5000888672765456	0.5004493751095680	0.4340875291859874
0.5003931899863190	0.5021035086169547	0.5852240094407435

REFERENCES

S1 B. Ravel, M. Newville, ATHENA, ARTEMIS, HEPHAESTUS: data analysis for X-ray absorption spectroscopy using IFEFFIT. *J. Synchrotron Radiat.* **12**, 537–541 (2005).

S2 T. Ben, H. Ren, S. Q. Ma, D. P. Cao, J. H. Lan, X. F. Jing, W. C. Wang, J. Xu, F. D., J. M. Simmons, S. L. Qiu, G. G. Zhu, *Angew. Chem., Int. Ed.* **2009**, *48*, 9457.

S3 C. Zhang, L. H. Peng, B. Y. Li, Y. Liu, P. C. Zhu, Z. Wang, D. H. Zhan, B. Tan, X. L. Yang, H. B. Xu, *Polym. Chem.* **2013**, *4*, 3663.

S4 U. Beser, M. Kastler, A. Maghsoumi, M. Wagner, C. Castiglioni, M. Tommasini, A. Narita, X. L. Feng, K. Mu¨llen, *J. Am. Chem. Soc.* **2016**, *138*, 4322.

S5 R. Dovesi, R. Orlando, A. Erba, C. M. Zicovich-Wilson, B. Civalleri, S. Casassa, L. Maschio, M. Ferrabone, M. D. L. Pierre, P. DArco, Y. No€el, M. Causa, M. Rerat, B. Kirtman, *Int. J. Quantum Chem.* **2014**, *114*, 1287.

S6 M. D. L. Pierre, P. Karamanis, J. Baima, R. Orlando, C. Pouchan, R. Dovesi, *J. Phys. Chem. C.* **2013**, *117*, 2222.

S7 G. Kresse, J. Hafner, *Phys. Rev. B.* **1994**, *49*, 14251.

S8 G. Kresse, J. Furthmüller, *Phys. Rev. B.* **1996**, *54*, 11169.

S9 J. P. Perdew, K. Burke, M. Ernzerhof, Generalized gradient approximation made simple. *Phys. Rev. Lett.* **1996**, *77*, 3865.

S10 G. Kresse, D. Joubert, *Phys. Rev. B.* **1999**, *59*, 1758.

S11 S. Lebe`gue, J. Harl, Tim Gould, J. G. A´ ngya´n, G. Kresse, J. F. Dobson, *Phys. Rev. Lett.* **2010**, *105*, 196401.

S12 S. Grimme, J. Antony, S. Ehrlich, H. Krieg, *J. Chem. Phys.* **2010**, *132*, 154104.

S13 G. Henkelman, A. Arnaldsson, H. Jónsson, *Comput. Mater. Sci.* **2006**, *36*, 354.

S14 S. Maintz, V. L. Deringer, A. L. Tchougréeff, R. Dronskowski, *J. Comput. Chem.* **2013**, 34, 2557.

S15 S. Maintz, V. L. Deringer, A. L. Tchougréeff, R. Dronskowski, *J. Comput. Chem.* **2016**, 37, 1030.

S16 Y. P. Wu, W. Zhou, J. Zhao, W. W. Dong, Y. Q. Lan, D. S. Li, C. H. Sun, X. H. Bu, *Angew. Chem., Int. Ed.* **2017**, *56*, 13001.

S17 G. Gao, A. P. O'Mullane, A. Du, *ACS Catal.* **2017**, **7**, 494.

S18 J. Yao, Z. Zheng, G. Yang, *Adv. Funct. Mater.* **2017**, *27*, 1701823.

S19 W. S. Li, Z. Guo, L. T. Jiang, L. Zhong, G. N. Li, J. J. Zhang, K. Fan, S. Gonzalez-Cortes, K. J. Jin, C. J. Xu, T. C. Xiao, P. P. Edwards, *Chem. Sci.* **2020**, *11*, 2716.

S20 D. B. Liu, X. Y. Li, S. M. Chen, H. Yan, C. D. Wang, C. Q. Wu, Y. A. Haleem, S. Duan, J. L. Lu, B. H. Ge, P. M. Ajayan, Y. Luo, J. Jiang, L. Song, *Nat. Energy.* **2019**, *4*, 512.

S21 K. Jiang, S. Back, A. J. Akey, C. Xia, Y. F. Hu, W. T. Liang, D. Schaak, E. Stavitski, J. K. Nørskov, S. Siahrostami, H. T. Wang, *Nat. Commun.* **2019**, *10*, 3997.

S22 H. B. Zhang, P. F. An, W. Zhou, B. Y. Guan, P. Zhang, J. C. Dong, X. W. Lou, *Sci. Adv.* **2018**, *4*, eaao6657.

S23 K. Li, Y. Li, Y. M. Wang, J. J. Ge, C. P. Liu, W. Xing, *Energy Environ. Sci.* **2018**, *11*, 1232.

S24 J. R. Yang, W. H. Li, S. D. Tan, K. N. Xu, Y. Wang, D. S. Wang, Y. D. Li, *Angew. Chem. Int. Ed*. **2021**, 60, 19085–19091
